# Normative values for lung, bronchial sizes, and bronchus-artery ratios in chest CT scans: from infancy into young adulthood

**DOI:** 10.1007/s00330-025-11367-w

**Published:** 2025-02-01

**Authors:** Qianting Lv, Yuxin Chen, Daan Caudri, Eleni-Rosalina Andrinopoulou, Wieying Kuo, Jean-Paul Charbonnier, Robert J. Fleck, Luis Riera Soler, Matteo Paoletti, Francois Vermeulen, Giovanni Morana, Edward Y. Lee, Marleen de Bruijne, Harm A. W. M. Tiddens, Pierluigi Ciet, for the Normal Chest CT Study Group, Matteo Paoletti, Francois Vermeulen, Giovanni Morana, Edward Y. Lee, Lauren Akesson, Silvia Bertolo, Alan S. Brody, Kris de Boeck, Pim A. de Jong, Robert J. Fleck, Francesco Fraioli, Pilar Garcia-Peña, Silvia Gartner, Anders Lindblad, Michael McCartin, Christian P. Mol, Arlette E. Odink, Stephen M. Stick

**Affiliations:** 1 Department of Paediatric Pulmonology and Allergology, Erasmus Medical Centre - Sophia Children’s Hospital, Rotterdam, The Netherlands; 2 Department of Radiology and Nuclear Medicine, Erasmus MC, Rotterdam, The Netherlands; 3 Department of Biostatistics, Erasmus MC, Rotterdam, The Netherlands; 4 Department of Epidemiology, Erasmus MC, Rotterdam, The Netherlands; 5 Voiant Clinical, Waltham, MA USA; 6 Thirona, Nijmegen, The Netherlands; 7 Department of Radiology, Cincinnati Children’s Hospital Medical Center and University of Cincinnati, Cincinnati, OH US; 8 Department of Pediatric Radiology, Hospital Universitari Vall d´Hebron, Barcelona, Spain; 9 Department of Advanced Imaging and Artificial Intelligence, IRCCS Mondino Foundation, Pavia, Italy; 10 Department of Pediatrics, University of Leuven, Leuven, Belgium; 11 Department of Radiology, Ca’ Foncello General Hospital, Treviso, Italy; 12 Department of Radiology, Boston Children’s Hospital and Harvard Medical School, Boston, MA USA; 13 Department of Computer Science, University of Copenhagen, Copenhagen, Denmark; 14 Department of Radiology, University of Cagliari, Cagliari, Italy; 15 Department of Respiratory Medicine, Princess Margaret Hospital for Children and Telethon Kids Institute, Perth, Australia; 16 Department of Radiology, Cincinnati Children’s Hospital, University of Cincinnati College of Medicine, Cincinnati, OH US; 17 Department of Radiology, University Medical Center Utrecht, Utrecht, The Netherlands; 18 Department of Radiology, Cincinnati Children’s Hospital Medical Center, Cincinnati, OH US; 19 Institute of Molecular Imaging, University College London, London, UK; 20 Department of Paediatric Pulmonology, Hospital Universitari Vall d´Hebron, Barcelona, Spain; 21 Department of Pediatrics, Queen Silvia Children’s Hospital, University of Gothenburg, Gothenburg, Sweden; 22 Image Sciences Institute, University Medical Center Utrecht, Utrecht, The Netherlands; 23 Department of Radiology, Erasmus University Medical Center, Rotterdam, The Netherlands

**Keywords:** Adolescents, Bronchi, Computer-assisted diagnosis, Reference values

## Abstract

**Objective:**

To estimate the developmental trends of quantitative parameters obtained from chest computed tomography (CT) and to provide normative values on dimensions of bronchi and arteries, as well as bronchus-artery (BA) ratios from preschool age to young adulthood.

**Materials and methods:**

Two independent radiologists screened a dataset of 1160 chest CT scans, initially reported as normal, from participants aged 0 to 24 years. Using an automated deep learning-based algorithm, we computed the following bronchus and artery parameters: bronchial outer diameter (B_out_), bronchial inner diameter (B_in_), adjacent pulmonary artery diameter (A), bronchial wall thickness (B_wt_), bronchial wall area (B_WA_), and bronchial outer area (B_OA_). From these parameters, we computed the following ratios: B_out_/A, B_in_/A, B_wt_/A, B_wt_/B_out_, and B_WA_/B_OA_. Furthermore, mean lung density, total lung volume, and the square root of wall area of bronchi with a 10-mm lumen perimeter (Pi10) were obtained. The effects on CT parameters of age, sex, and iodine contrast were investigated using mixed-effects or regression model analyses.

**Results:**

375 normal inspiratory chest CT scans (females / males = 156 / 219; mean age [SD] 12.7 [5.0] years) met the inclusion criteria. B_out_ and B_in_ progressively increased with age (all *p* < 0.05), but B_wt_, B_out_/A, B_in_/A, B_wt_/A, B_wt_/B_out_, or B_WA_/B_OA_ did not. Total lung volume and mean lung density continuously increased with age (both *p* < 0.001), while Pi10 did not exhibit such a trend. B_out_, total lung volume, and mean lung density were the only parameters that differed between males and females, all higher in males than females (all *p* < 0.03). The presence of iodinated contrast led to greater values for B_wt_, B_wt_/B_out_, and B_WA_/B_OA_, but lower values for B_in_, B_out_/A, B_in_/A, and B_wt_/A (all *p* < 0.01).

**Conclusion:**

Quantitative CT parameters of both lung parenchyma and bronchi exhibit growth-related changes, but from 6 to 24 years ratios between bronchus and artery dimensions remain constant. Contrast-enhanced CT scans affect the assessment of lung parenchyma and bronchial size. We propose age and technique-dependent normative values for bronchial dimensions and wall thickness.

**Key Points:**

***Question*** *What are the developmental trends of quantitative lung CT parameters in patients from childhood into young adulthood?*

***Findings*** *The ratio between bronchus and pulmonary artery dimensions demonstrates consistent values across age groups, indicating synchronized growth between bronchi and paired pulmonary arteries.*

***Clinical relevance*** *Our findings highlight the importance of standardized CT protocol and volume acquisition, and emphasize the need for ongoing collection of normal chest CT scans to refine the proposed reference values.*

## Introduction

Airway disease is a major component of a wide range of lung disorders in childhood and young adulthood. Chest computed tomography (CT) is currently the most sensitive imaging method for evaluating structural airway changes, because of its high resolution and inherent air-lung tissue contrast [1]. Important airway-related changes routinely evaluated on chest CT scans are bronchial widening and bronchial wall thickening, which are key features for the diagnosis of bronchiectasis [2].

In clinical practice, for assessing bronchial widening and wall thickening, radiologists typically use visual estimation, comparing bronchus dimensions with adjacent pulmonary arteries [2]. Nonetheless, this method has limitations. First, the radiologist might find it difficult to discern subtle changes or to evaluate alterations in the smaller airways. Second, it lacks quantitative data necessary to precisely measure the severity and extent of the bronchial widening and wall thickening. Additionally, variability between and within observers in defining airway disease severity poses challenges [3].

Ideally, precise objective measurements are pivotal, a practice that has been realized in only a limited number of studies. For instance, in cystic fibrosis, manual measurements of all visible pairs of bronchus-artery (BA) dimensions on chest CT scans proved sensitive and accurate in identifying and tracking bronchial wall widening and thickening [4]. However, this method is time-consuming, taking one to five days to measure all BA pairs on a chest CT scan depending on patient age and size. Recently, an automated algorithm was developed and validated, reducing analyzing time to 20 min and enabling comprehensive analysis of bronchus and pulmonary artery dimensions for all visible BA pairs on chest CT scans [5].

A recent study provided normative data on trachea, right and left main bronchi dimensions obtained from CT scans, focusing on children and adolescents [6]. However, there is limited data on BA dimensions of smaller airways serving as reference values. This issue was addressed in a systematic review by Meerburg et al, in which the authors concluded that no validation study exists for establishing cutoff values defining bronchiectasis and that BA ratios could depend on age [7]. Additionally, a prospective assessment of bronchial dimensions in healthy subjects using chest CT would involve exposing them to ionizing radiation, raising ethical concerns, especially in children.

Therefore, this study aimed to assess the developmental trend of quantitative CT parameters derived from chest CT scans and establish normative data of lung density, BA dimensions and BA ratios based on a large cohort of normal chest CT scans obtained from participants from childhood into young adulthood.

## Materials and methods

### Study participants

This retrospective study included participants who underwent chest CT between 2003 and 2013 for various diagnostic indications. CT scans were collected in an anonymized imaging archive from 10 different centers across America and Europe as part of the international multicenter Normal Chest CT Study Group (NCCTSG) to establish reference values of central airways. In the previous study, inclusion criteria were: all chest CT protocols; age 0 and 20 years at the time of CT; and normal trachea, lung, and heart as indicated by the radiologist reports. 294 of the 1160 patients have been previously reported [6]. The previous article provided normative data on trachea and right and left main bronchi dimensions of children and adolescents. The current manuscript presents normative data for lung density, BA dimensions, BA ratios from segmental bronchi to the most peripheral detectable bronchi.

The study was approved by the institutional review board of all participating centers (listed in Appendix S1).

### CT data

Each CT is anonymous and provided with a code (study number). The study number consists of a center number followed by a patient number. Anonymized CT scans contained only information about sex, age at the time of the chest CT, and CT protocol. Information related to the indication for the chest CT scan, comorbidities, and the patient’s weight, height, and ethnicity were not available in relation to the ethics approval. Initial reports by radiologists at international centers indicated normal findings for these scans. For the previous study, an independent thoracic radiologist with 20 years of experience in cardiothoracic imaging (observer 1) evaluated the chest CT scan findings for image quality and normal findings. A second observer, with 3 years of experience in thoracic imaging (observer 2), reevaluated all CT scans to assess image quality, lung inflation, and normal findings. The eligibility criteria for chest CT scans included: no evidence of pulmonary or cardiac abnormalities (including the airways, vessels, lung parenchyma and chest wall); contiguous helical CT acquisition; full lung coverage, slice thickness of 1.5 mm or less; detection of more than 10 bronchus-artery pairs; both non-contrast and contrast-enhanced scans; and absence of significant motion artifacts. In addition, statistical analysis excluded participants under 7 years as CT scans in these participants were more likely to be obtained during a voluntary inspiratory breath hold. Lung inflation assessment to determine the quality of inspiratory level as good, moderate, or poor was conducted using a Standard Operating Procedure detailed in Appendix S2 [8]. Summary of the scan parameters is shown in Tables S1a and S1b.

### Quantitative analysis

**Fig. 1:**
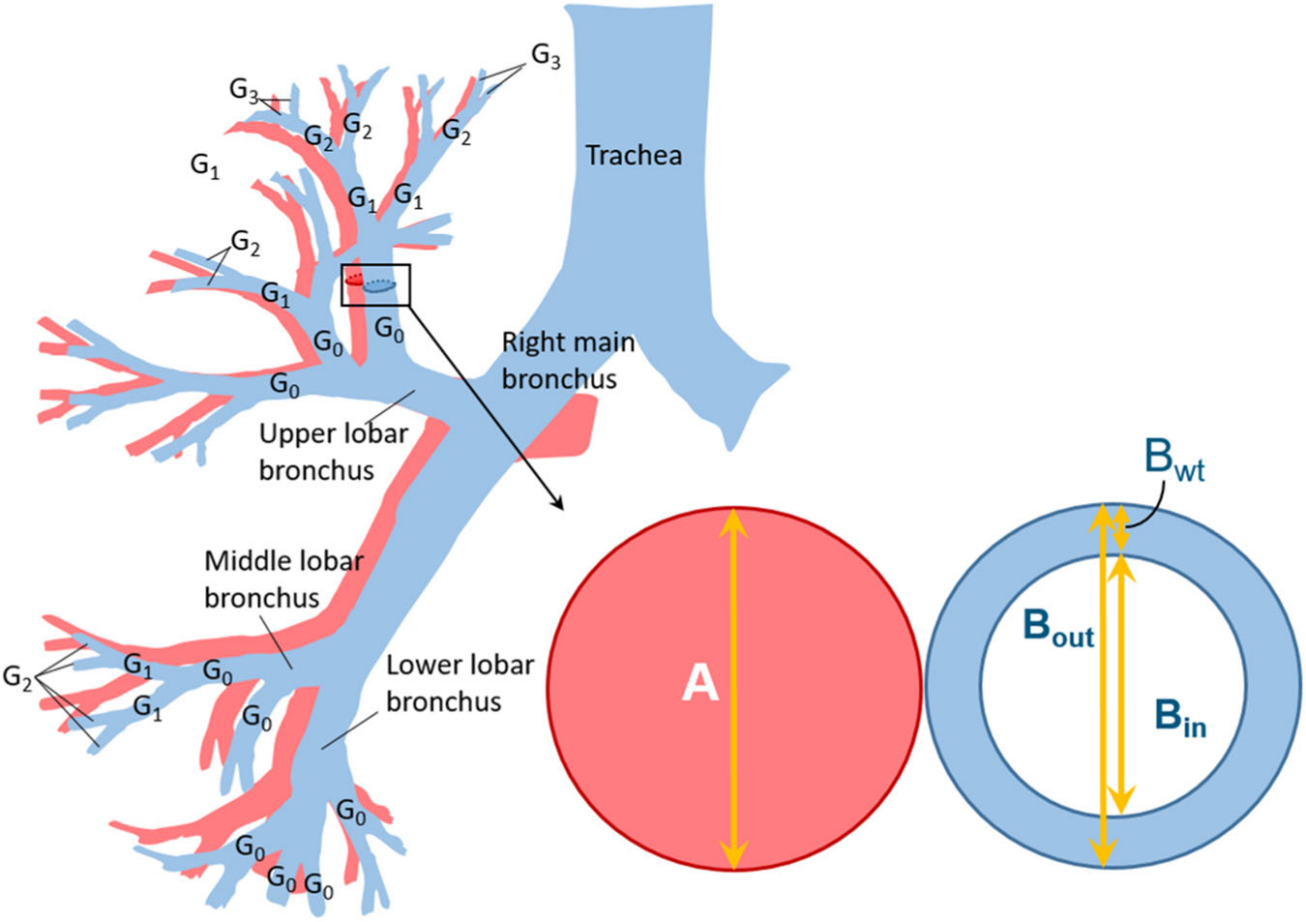
Schematic view of the bronchial tree and of a bronchus-artery (BA) pair in cross-section, showing measurements for each bronchus [5]. The bronchial tree (blue) with its accompanying pulmonary artery system (pink) is shown on the left. The segmental bronchi are defined as G_0_ and the sub-segmental bronchi as G_1_. When a bronchus splits into two or more branches, the generation number increases by one. On the right, a bronchus-artery (BA)-pair is shown, the bronchus in blue and the adjacent pulmonary artery in pink, which is drawn by pretending they are perfect circles. The arrows depict the bronchus and pulmonary artery dimensions that can be measured by the automatic BA method: Bronchial outer diameter (B_out_); Bronchial inner diameter (B_in_), Bronchial wall thickness (B_wt_), and adjacent pulmonary Artery diameter (A)

The BA method was executed using LungQ-BA V2.0.1 (Thirona) software. The LungQ-BA software automatically identifies patient-specific anatomical features from chest CT images [5]. Briefly, the BA method [9] utilized two AI-based algorithms on the inspiratory CT scans. The first algorithm segments the bronchial tree from the CT images focusing on bronchi with a visible lumen. The second algorithm matches the adjacent artery for each identified bronchial branch (BA pair), quantifying several parameters measured perpendicular to the longitudinal axis of the bronchus and pulmonary artery. These parameters include bronchial outer diameter (B_out_), bronchial inner diameter (B_in_), bronchial wall thickness (B_wt_), diameter of the adjacent pulmonary artery (A) (Fig. 1). The following ratios are computed for each BA pair: B_out_/A, B_in_/A, and B_wt_/A. The BA dimensions of each individual bronchial branch are computed as averages of all measurements within that branch. For each BA pair, the bronchial generation number (G) is defined starting from segmental bronchi (G_0_), and incrementally numbered as bronchi bifurcate into two or more bronchial branches (Fig. 1). Labels automatically extend from segmental bronchi to the most peripheral detectable bronchi, registering additional information, such as segmental generation (generation number), bronchopulmonary segments, and lobe. The B_wt_/B_out_, and B_WA_/B_OA_ (bronchial wall area/bronchial outer wall area, B_OA_ = [B_out_/2]^2^ × π, B_WA_ = [B_out_/2]^2^ × π − [B_in_/2]^2^ × π) are computed using the assumption of a perfect circle for bronchial cross-sections. The square root of wall area of a 10-mm lumen perimeter (Pi10), a parameter used in various studies to evaluate bronchial wall thickness, total lung volume, and mean lung density, is automatically derived by using another module from the same software package.

### Interrater reliability

To test inter-observer reliability of observer 1 and 2 for normal findings of CT scans, Cohen’s kappa statistic is used. The Cohen’s kappa result should be interpreted as follows: values ≤ 0 as indicating no agreement and 0.01–0.20 as none to slight, 0.21–0.40 as fair, 0.41–0.60 as moderate, 0.61–0.80 as substantial, and 0.81–1.00 as almost perfect agreement [10].

### Statistical analysis

Participants were divided into seven age subgroups: six subgroups with 3-year intervals for children and one separate group for young adults (18–24 years). CT parameters are reported as either the median with interquartile range (IQR) or the mean ± standard deviation (SD), depending on the data distribution. The threshold for ratios to differentiate abnormal from normal bronchi was chosen as the [mean + 2SD] [11]. For descriptive analysis, we included all seven age subgroups. However, to investigate the effect of contrast on BA dimensions and ratios, we included the use of iodine contrast in all models.

Mixed-effects models were used to analyze bronchus diameters (B_out_, B_in_, and B_wt_) and ratios (B_out_/A, B_in_/A, B_wt_/A, and B_wt_/B_out_, B_WA_/B_OA_) with the assumption of multiple measurements per subject. Age, sex, total lung volume, CT scanners (Philips, SIEMENS, and TOSHIBA), and use of iodine contrast (yes/no) were considered as fixed effects. Participants, lobes, and segmental generations were treated as random effects in the analyses. For outcomes (B_wt_, B_out_/A, B_in_/A, B_wt_/A, and B_wt_/B_out_), the logarithmic scale was used due to non-normally distributed residuals. Linear regression models’ analyses were used to assess the relationship between mean lung density, total lung volume, Pi10, age, sex, CT scanners (Philips, SIEMENS, and TOSHIBA), and the use of iodine contrast.

All statistical analyses were performed using R, version 4.3.2 (R Foundation for Statistical Computing, 2005). A *p*-value less than 0.05 was considered significant. Adjustment for multiple testing [12] was not performed and the estimate, confidence interval and unadjusted *p*-values are provided for data interpretation.

## Results

### Study population

**Fig. 2:**
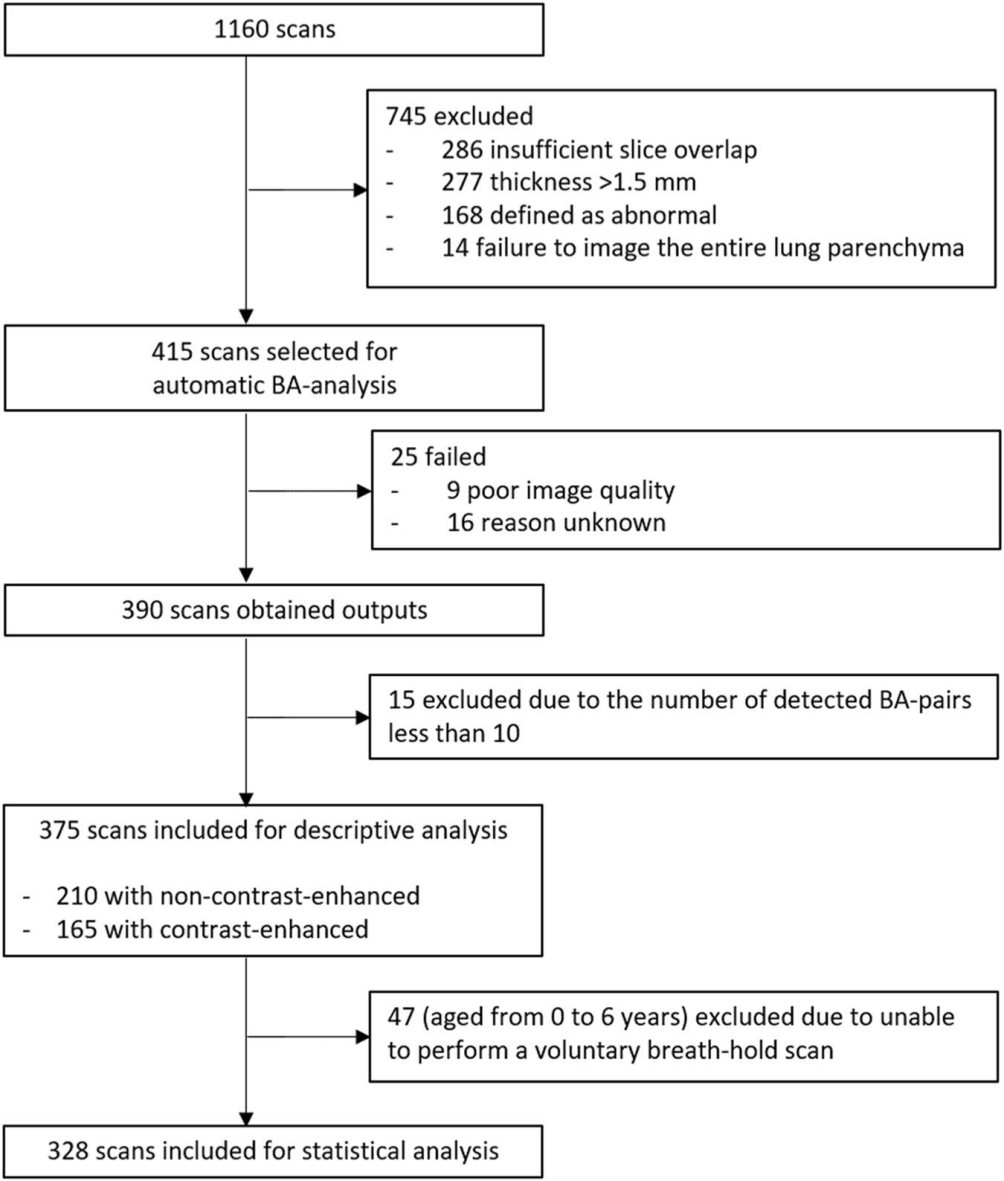
Flowchart of Normal Chest CT Group dataset

From the original dataset of 1160 CT scans from the Normal Chest CT Group, 745 scans were excluded for either not meeting the inclusion criteria or being unsuitable for analysis with the automatic BA method. Of the remaining 415 scans, an additional 35 were excluded due to analysis failures in 25 scans and the detection of fewer than 10 BA pairs in 15 subjects. A detailed flowchart illustrating this process is shown in Fig. 2. The final dataset comprised 375 CT scans, consisting of 156 females and 219 males (mean age (SD) 12.7 (5.0) years; range 0–24 years). The agreement between observers on normal findings of CT scans was fair (0.32; 95% CI 0.23 to 0.41). Iodine contrast was administered in 165 CT scans (44%), while 210 were non-contrast CT scans. 96% of the CT scans had moderate to good lung inflation. The characteristics of the analyzed CT scans are presented in Table 1.

**Table 1:** Demographics of Normal Chest CT datasets and number of BA pairs assessed by automatic BA method

Age range (years)	Sample size (CT with contrast)	Age (years) Median [IQR range]	Male (%)	No. of BA pairs in total	No. of BA pairs per scan Mean (SD)	Range of detectable generations
All	375 (165)	13.4 [9.9, 16.8]	58.4	45,122	120 (68)	G_0_–G_4_
> 0 to ≤ 3	24 (6)	1.6 [1.4, 2.3]	62.5	753	31 (18)	G_0_–G_4_
> 3 to ≤ 6	23 (5)	4.7 [3.8, 5.3]	69.6	1037	45 (31)	G_0_–G_6_
> 6 to ≤ 9	34 (10)	7.6 [6.7, 8.3]	38.2	2962	87 (43)	G_0_–G_6_
> 9 to ≤ 12	71 (29)	10.8 [10.0, 11.4]	62.0	8531	120 (45)	G_0_–G_7_
> 12 to ≤ 15	82 (28)	13.6 [12.8, 14.5]	68.3	10,932	133 (56)	G_0_–G_8_
> 15 to ≤ 18	85 (48)	16.7 [15.8, 17.4]	49.4	11,426	134 (69)	G_0_–G_8_
> 18 to ≤ 24	56 (39)	19.0 [18.5, 19.6]	58.9	9481	169 (74)	G_0_–G_9_

*IQR* interquartile range (shows in (25th percentile, 75th percentile)), *%* percentage, *no*. number, *SD* standard deviation, *G* segmental generation, *G*_*0*_ segmental bronchi, *G*_*1*_ first consecutive sub-segmental bronchi, and so forth

**Fig. 3:**
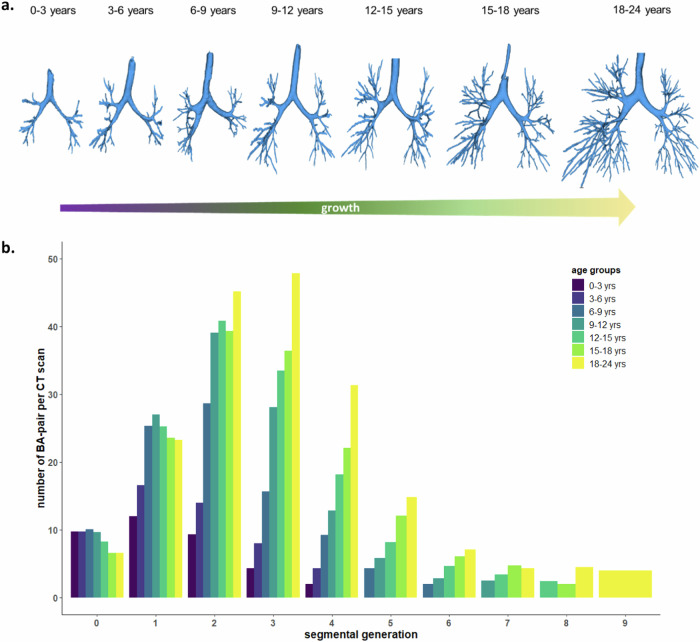
Relationship between branching and age. BA pair, bronchus-artery pair; CT, computed tomography. **a** Representative three-dimensional volume rendering of the bronchial tree for each age group. **b** Plot of the average BA-pair count compared to the segmental generations. In the horizontal axis of Fig. 2b, segmental generation 0 indicates the segmental bronchi and 1 indicates consecutive sub-segmental bronchi and so forth. The number of BA pairs represents the number of detectable bronchial branches. Note that according participant’s group, bronchi located more distally from segmental generation 4 are present in lower numbers or not visible, especially in the younger age groups, due to the resolution of the CT scanner. Some adjacent arteries originating from G_0_ were challenging to detect due to their clustered anatomical structure. Thus unpaired bronchial measurements (lacking paired arteries) were excluded from the dataset. As a result, the average number of branching artery pairs from G_0_ ranged between 7 to 10

From the 375 CT scans that were successfully analyzed by the BA method, more generations and BA pairs could be detected with increasing age. The three-dimensional reconstructions of the bronchial tree (Fig. 3a) and the plot illustrating the average BA-pair count with the segmental generations (Fig. 3b) clearly demonstrate the growth-related increase in detectable BA pairs across patients. The boxplot of BA-pair number with segmental generations in each age group is provided in Appendix Fig. S1.

All measurements showed no difference among the three manufacturers (all *p* > 0.05). CT parameters assessed by the BA method on iodine contrast CT scans differed from those on non-contrast CT scans, therefore results are shown separately.

### Non-contrast CT scan results

#### Bronchial dimensions

**Fig. 4:**
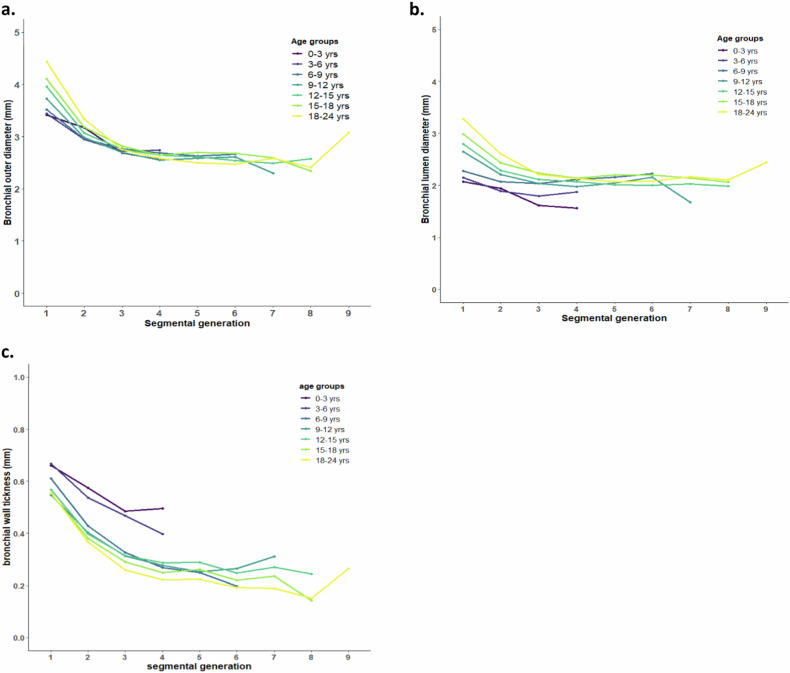
Bronchial dimensions of non-iodine contrast CT scans in each segmental generation grouped by age. Plots (**a**–**c**) show trends of B_out_ (**a**), B_in_ (**b**), and B_wt_ (**c**) (vertical axis) in each age group against segmental generation (horizontal axis). Note that 1 indicates the sub-segmental bronchi and G_n+1_ the consecutive higher generations. Each line represents a different age group. The point on each line shows the median of the bronchial dimensions in each segmental generation. The variability of the median of bronchial dimensions after segmental generation 6 is caused by the limited number of detected BA pairs related to the resolution limitation of commonly used CT scanners

Figure 4 shows the measurements obtained by the automatic BA method, showing that the size of B_out_, B_in_, and B_wt_ decreased from the central bronchi towards the periphery of the lung in each age group. Median and IQR of the B_out_, B_in_, and B_wt_ according to age group are presented in Table S2. Mixed-model analyses showed an increase of 0.02 mm per additional year of age in both B_out_ (95% CI 0.006 to 0.034; *p* = 0.005) and B_in_ (95% CI 0.004 to 0.027 mm; *p* = 0.007), while B_wt_ did not change with age (estimate 0.003 mm; 95% CI −0.024 to 0.029; *p* = 0.18). The B_out_ (95% CI −0.179 to −0.012 mm; *p* = 0.03) of female was 0.1 mm less than male but there were no differences in B_in_ (estimate −0.03 mm; 95% CI −0.094 to 0.042; *p* = 0.46) or B_wt_ (estimate 0.03 mm; 95% CI 0.002 to 0.048; *p* = 0.09) by sex (Table S3).

#### Bronchial ratios

**Fig. 5:**
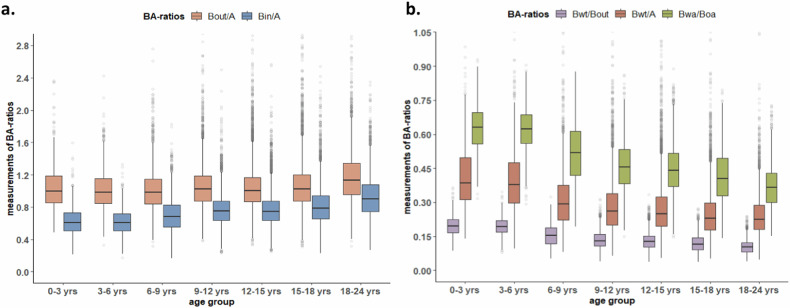
The BA ratios of non-iodine contrast CT scans in each age group. Panel **a** shows the ratios of bronchial diameters, while Panel **b** represents the ratios of bronchial wall thickness. B_out_/A: ratio of bronchial outer diameter and adjacent pulmonary artery; B_in_/A: ratio of bronchial inner diameter and adjacent pulmonary artery; B_wt_/A: ratio of bronchial wall thickness and adjacent pulmonary artery; B_wt_/B_out_: ratio of bronchial wall thickness and bronchial outer diameter; B_wa_/B_oa_: ratio of bronchial wall area and bronchial outer wall area. Each box shows median (horizontal line), interquartile range (solid box), 1.5*quartile range (whiskers), and outliers (circles). Plots show stable ratios of B_out_/A, B_in_/A, B_wt_/A, B_wt_/B_out_, and B_wa_/B_oa_ across the age groups

Figure 5 shows changes in BA ratios obtained using the automated BA method across age groups. Three bronchial wall-related ratios (B_wt_/A, B_wt_/B_out_, and B_WA_/B_OA_) were higher in the 0 to 6 years subgroups compared to age groups above 6 years (Fig. 4b). Median (IQR) and the upper limit of normal (shown as mean + 2SD) for five ratios (B_out_/A, B_in_/A, B_wt_/A and B_wt_/B_out_, B_WA_/B_OA_) in each segmental generation are presented per age group in Table 2. After adjusting for age, sex, total lung volume, and use of iodine contrast, no changes were observed in B_out_/A (estimate 0.001/year; 95% CI −0.038 to 0.041; *p* = 0.67), B_in_/A (estimate 0.002/year; 95% CI −0.036 to 0.040; *p* = 0.50), B_wt_/A (estimate 0.001/year; 95% CI −0.007 to 0.008; *p* = 0.31), B_wt_/B_out_ (estimate 0.0004/year; 95% CI −0.007 to 0.008; *p* = 0.46), or B_WA_/B_OA_ (estimate 0.0003/year; 95% CI −0.003 to 0.004; *p* = 0.86) in relation to participant age. No sex differences were found for all five ratios (B_out_/A: estimate −0.004, 95% CI −0.034 to 0.026, *p* = 0.80; B_in_/A: estimate −0.016, 95% CI −0.045 to 0.013, *p* = 0.29; B_wt_/A: estimate 0.004, 95% CI −0.002 to 0.009, *p* = 0.25; and B_wt_/B_out_: estimate 0.005, 95% CI −0.002 to 0.011, *p* = 0.17; and B_WA_/B_OA_: estimate −0.016, 95% CI −0.037 to 0.004, *p* = 0.11) (Table S4).

**Table 2:** The median (IQR) and threshold of the BA ratios of non-iodine contrast (*n* = 210) CT scans in each segmental generation divided by age group

B_out_/A	0–3 years (*n* = 18)	3–6 years (*n* = 18)	6–9 years (*n* = 24)	9–12 years (*n* = 42)	12–15 years (*n* = 54)	15–18 years (*n* = 37)	18–24 years (*n* = 17)
G_1_	1.02 (0.88, 1.21)	1.00 (0.89, 1.18)	1.01 (0.87, 1.15)	1.06 (0.92, 1.22)	1.06 (0.93, 1.25)	1.07 (0.94, 1.27)	1.19 (1.04, 1.39)
G_2_	0.95 (0.84, 1.08)	0.95 (0.82, 1.07)	0.96 (0.83, 1.13)	1.02 (0.87, 1.16)	0.98 (0.86, 1.14)	1.02 (0.87, 1.19)	1.12 (0.97, 1.32)
G_3_	0.85 (0.78, 0.98)	0.80 (0.67, 0.91)	0.95 (0.80, 1.11)	0.99 (0.84, 1.14)	0.97 (0.84, 1.12)	1.00 (0.85, 1.18)	1.11 (0.93, 1.30)
G_4_	-	-	0.93 (0.81, 1.07)	0.94 (0.79, 1.09)	0.97 (0.84, 1.11)	0.99 (0.85, 1.15)	1.13 (0.94, 1.37)
G_5_	-	-	-	-	0.94 (0.82, 1.11)	0.96 (0.82, 1.11)	1.11 (0.94, 1.30)
G_6_	-	-	-	-	-	-	0.97 (0.81, 1.19)
Threshold							
Mean ± [SD]	1.03 ± [0.28]	0.98 ± [0.25]	1.00 ± [0.26]	1.03 ± [0.27]	1.03 ± [0.26]	1.06 ± [0.30]	1.17 ± [0.31]
Mean + 2SD	1.58	1.49	1.51	1.57	1.55	1.66	1.79
**B**_**in**_**/A**	**0–3 years**	**3–6 years**	**6–9 years**	**9–12 years**	**12–15 years**	**15–18 years**	**18–24 years**
G_1_	0.65 (0.51, 0.76)	0.63 (0.53, 0.73)	0.66 (0.55, 0.79)	0.76 (0.65, 0.88)	0.76 (0.65, 0.89)	0.78 (0.67, 0.95)	0.90 (0.77, 1.05)
G_2_	0.59 (0.50, 0.71)	0.60 (0.51, 0.70)	0.68 (0.55, 0.82)	0.75 (0.63, 0.87)	0.73 (0.62, 0.85)	0.77 (0.65, 0.93)	0.88 (0.74, 1.04)
G_3_	0.56 (0.45, 0.65)	0.52 (0.43, 0.62)	0.71 (0.56, 0.84)	0.74 (0.62, 0.87)	0.74 (0.63, 0.86)	0.79 (0.64, 0.94)	0.90 (0.74, 1.07)
G_4_	-	-	0.70 (0.56, 0.88)	0.72 (0.59, 0.84)	0.75 (0.64, 0.88)	0.78 (0.65, 0.94)	0.93 (0.75, 1.15)
G_5_	-	-	-	-	0.73 (0.63, 0.88)	0.77 (0.64, 0.90)	0.90 (0.77, 1.09)
G_6_	-	-	-	-	-	-	0.81 (0.65, 1.00)
Threshold							
Mean ± [SD]	0.63 ± [0.18]	0.61 ± [0.16]	0.70 ± [0.21]	0.76 ± [0.21]	0.76 ± [0.21]	0.81 ± [0.25]	0.93 ± [0.27]
Mean + 2SD	0.98	0.93	1.12	1.18	1.18	1.31	1.46
**B**_**wt**_**/A**	**0–3 years**	**3–6 years**	**6–9 years**	**9–12 years**	**12–15 years**	**15–18 years**	**18–24 years**
G_1_	0.20 (0.15, 0.26)	0.19 (0.16, 0.24)	0.17 (0.14, 0.20)	0.15 (0.12, 0.19)	0.15 (0.12, 0.19)	0.14 (0.12, 0.18)	0.15 (0.12, 0.18)
G_2_	0.17 (0.14, 0.21)	0.16 (0.14, 0.20)	0.14 (0.11, 0.17)	0.13 (0.10, 0.16)	0.13 (0.10, 0.16)	0.12 (0.10, 0.15)	0.12 (0.10, 0.15)
G_3_	0.16 (0.14, 0.19)	0.13 (0.11, 0.17)	0.11 (0.09, 0.15)	0.11 (0.09, 0.15)	0.11 (0.09, 0.14)	0.10 (0.08, 0.13)	0.10 (0.09, 0.13)
G_4_	-	-	0.10 (0.08, 0.13)	0.10 (0.08, 0.13)	0.11 (0.08, 0.13)	0.09 (0.08, 0.12)	0.10 (0.08, 0.12)
G_5_	-	-	-	-	0.10 (0.08, 0.13)	0.09 (0.07, 0.12)	0.10 (0.08, 0.12)
G_6_	-	-	-	-	-	-	0.08 (0.07, 0.10)
Threshold							
Mean ± [SD]	0.20 ± [0.08]	0.19 ± [0.07]	0.15 ± [0.06]	0.14 ± [0.06]	0.13 ± [0.05]	0.12 ± [0.05]	0.12 ± [0.04]
Mean + 2SD	0.36	0.33	0.27	0.25	0.23	0.22	0.21
**B**_**wt**_**/B**_**out**_	**0–3 years**	**3–6 years**	**6–9 years**	**9–12 years**	**12–15 years**	**15–18 years**	**18–24 years**
G_1_	0.20 (0.16, 0.22)	0.19 (0.17, 0.22)	0.17 (0.14, 0.20)	0.14 (0.12, 0.17)	0.14 (0.12, 0.17)	0.13 (0.11, 0.16)	0.13 (0.11, 0.14)
G_2_	0.19 (0.16, 0.21)	0.18 (0.15, 0.21)	0.15 (0.12, 0.18)	0.13 (0.11, 0.16)	0.13 (0.11, 0.15)	0.12 (0.10, 0.15)	0.11 (0.09, 0.13)
G_3_	0.18 (0.16, 0.22)	0.17 (0.14, 0.20)	0.12 (0.10, 0.17)	0.12 (0.10, 0.14)	0.12 (0.10, 0.14)	0.10 (0.08, 0.13)	0.10 (0.08, 0.12)
G_4_	-	-	0.11 (0.09, 0.15)	0.11 (0.09, 0.13)	0.11 (0.09, 0.13)	0.10 (0.08, 0.12)	0.09 (0.07, 0.11)
G_5_	-	-	-	-	0.11 (0.09, 0.13)	0.10 (0.08, 0.12)	0.09 (0.08, 0.10)
G_6_	-	-	-	-	-	-	0.08 (0.07, 0.09)
Threshold							
Mean ± [SD]	0.19 ± [0.04]	0.19 ± [0.04]	0.15 ± [0.05]	0.13 ± [0.04]	0.13 ± [0.04]	0.12 ± [0.04]	0.10 ± [0.03]
Mean + 2SD	0.28	0.27	0.24	0.20	0.20	0.19	0.16
**B**_**WA**_**/B**_**OA**_	**0–3 years**	**3–6 years**	**6–9 years**	**9–12 years**	**12–15 years**	**15–18 years**	**18–24 years**
G_1_	0.63 (0.55, 0.70)	0.63 (0.58, 0.68)	0.57 (0.49, 0.64)	0.49 (0.43, 0.55)	0.49 (0.43, 0.56)	0.46 (0.40, 0.54)	0.44 (0.38, 0.48)
G_2_	0.61 (0.54, 0.67)	0.60 (0.52, 0.66)	0.50 (0.41, 0.60)	0.46 (0.38, 0.53)	0.45 (0.38, 0.52)	0.42 (0.35, 0.50)	0.39 (0.33, 0.45)
G_3_	0.60 (0.53, 0.68)	0.56 (0.47, 0.64)	0.43 (0.35, 0.55)	0.42 (0.36, 0.49)	0.41 (0.35, 0.48)	0.37 (0.31, 0.46)	0.34 (0.29, 0.41)
G_4_	-	-	0.38 (0.32, 0.50)	0.40 (0.34, 0.46)	0.39 (0.33, 0.46)	0.35 (0.29, 0.43)	0.31 (0.26, 0.38)
G_5_	-	-	-	-	0.40 (0.33, 0.46)	0.34 (0.28, 0.42)	0.32 (0.28, 0.37)
G_6_	-	-	-	-	-	-	0.30 (0.25, 0.34)
Threshold							
Mean ± [SD]	0.62 ± [0.10]	0.60 ± [0.10]	0.50 ± [0.13]	0.45 ± [0.11]	0.44 ± [0.10]	0.41 ± [0.11]	0.37 ± [0.09]
Mean + 2SD	0.82	0.80	0.76	0.66	0.64	0.62	0.54

The results of segmental generation G_1_ and higher are shown in median (IQR) and the threshold indicate bronchial widening or wall thickening if BA ratios greater than [mean + 2*SD] for all generations by age group. IQR, shown in (25th percentile, 75th percentile); G: segmental generations, and G_n+1_: the consecutive branching bronchi; B_out_/A: ratio between bronchial outer wall diameter and adjacent pulmonary artery; B_in_/A: ratio between bronchial inner wall diameter and the adjacent pulmonary artery; B_wt_/A: ratio between bronchial wall thickness and adjacent pulmonary artery diameter; B_wt_/B_out_: ratio between bronchial wall thickness and bronchial outer diameter; B_WA_/B_OA_: ratio between bronchial wall area and bronchial outer wall area

*IQR* interquartile range, *SD* standard deviation

#### Mean lung density, total lung volume, and Pi10

The median and IQR of the mean lung density, total lung volume, and Pi10 are shown according to age group in Table S5. After adjusting for age, sex, and use of iodine contrast, both mean lung density (estimate 7.7 Hounsfield Unit (HU) per year of age; 95% CI 5.20 to 10.24; *p* < 0.001) and total lung volume (estimate 235.4 mL per year; 95% CI 208.72 to 262.07; *p* < 0.001) increased with older age (Table S6). Conversely, Pi10 exhibited a decrease of 0.025 per year (95% CI −0.043 to −0.007; *p* = 0.006) with older age. Females had lower mean lung density (estimate −20.89 HU; 95% CI −35.256 to −6.524; *p* = 0.004) and total lung volume (estimate −786.36 mL; 95% CI −979.12 to −593.61; *p* < 0.001) compared to males (Table S6). However, there was no difference observed in Pi10 (estimate 0.012, 95% CI −0.086 to 0.109, *p* = 0.81) by sex. Both mean lung density (estimate −0.04 HU per mL; 95% CI −0.049 to −0.036; *p* < 0.001) and Pi10 (estimate −0.00006 per mL; 95% CI −0.000116 to −0.000001; *p* = 0.04) showed decrease with increasing total lung volume (Table S6).

### Iodine contrast CT scan results

Median (IQR) and the upper limit of normal (shown as mean + 2SD) for five ratios (B_out_/A, B_in_/A, B_wt_/A and B_wt_/B_out_, B_WA_/B_OA_) in each segmental generation are presented per age group in Table 3. The presence of iodinated contrast led to greater values for B_wt_ (estimate 0.04 mm; 95% CI 0.014 to 0.059; *p* = 0.007), B_wt_/B_out_ (estimate 0.01, 95% CI 0.006 to 0.019; *p* < 0.001), and B_WA_/B_OA_ (estimate 0.04, 95% CI 0.020 to 0.059; *p* < 0.001). Furthermore, it resulted in lower values for B_in_ (estimate −0.13 mm; 95% CI −0.198 to −0.069; *p* < 0.001), B_out_/A (estimate −0.22, 95% CI −0.254 to −0.188; *p* < 0.001), B_in_/A (estimate −0.17; 95% CI −0.199 to −0.143; *p* < 0.001), and B_wt_/A (estimate −0.01; 95% CI −0.019 to −0.008; *p* < 0.001) in comparison to non-iodine contrast scans. B_out_ (estimate −0.05 mm; 95% CI −0.132 to 0.026; *p* = 0.19) and Pi10 (estimate 0.007 mm; 95% CI −0.094 to 0.107; *p* = 0.90) were affected less in participants who received iodine contrast. The mean lung density of iodine contrast CT scans was higher (estimate 47.18 HU; 95% CI 33.346 to 61.014; *p* < 0.001) than that of non-iodine contrast scans. Detailed results of the BA method on iodine contrast CT scans are shown in the online supplement (Tables S3, S4, and S6).

**Table 3:** The median (IQR) and threshold of the BA ratios of iodine contrast (*n* = 165) CT scans in each segmental generation divided by age group

B_out_/A	0–3 years (*n* = 6)	3–6 years (*n* = 5)	6–9 years (*n* = 10)	9–12 years (*n* = 29)	12–15 years (*n* = 28)	15–18 years (*n* = 48)	18–24 years (*n* = 39)
G_1_	0.79 (0.68, 0.95)	0.74 (0.61, 0.90)	0.82 (0.66, 1.02)	0.83 (0.72, 1.01)	0.87 (0.75, 0.99)	0.88 (0.75, 1.06)	0.88 (0.74, 1.05)
G_2_	0.75 (0.62, 1.84)	0.72 (0.63, 0.83)	0.77 (0.63, 0.89)	0.77 (0.64, 0.94)	0.80 (0.68, 0.93)	0.85 (0.69, 1.02)	0.83 (0.69, 0.99)
G_3_	0.81 (0.71, 0.95)	0.67 (0.60, 0.77)	0.76 (0.62, 0.89)	0.75 (0.63, 0.94)	0.75 (0.64, 0.89)	0.87 (0.69, 1.06)	0.83 (0.67, 0.99)
G_4_	-	-	0.78 (0.69, 0.89)	0.74 (0.59, 0.91)	0.75 (0.63, 0.91)	0.91 (0.72, 1.13)	0.84 (0.69, 1.04)
G_5_	-	-	-	-	0.76 (0.61, 0.94)	0.94 (0.73, 1.17)	0.83 (0.68, 1.00)
G_6_	-	-	-	-	-	-	0.82 (0.70, 0.97)
Threshold							
Mean ± [SD]	0.80 ± [0.22]	0.75 ± [0.19]	0.80 ± [0.24]	0.82 ± [0.25]	0.83 ± [0.26]	0.92 ± [0.30]	0.88 ± [0.29]
Mean + 2SD	1.23	1.13	1.28	1.33	1.35	1.51	1.45
**B**_**in**_**/A**	**0–3 years**	**3–6 years**	**6–9 years**	**9–12 years**	**12–15 years**	**15–18 years**	**18–24 years**
G_1_	0.45 (0.36, 0.51)	0.42 (0.36, 0.50)	0.50 (0.39, 0.63)	0.57 (0.47, 0.70)	0.59 (0.50, 0.70)	0.60 (0.50, 0.71)	0.63 (0.52, 0.77)
G_2_	0.43 (0.36, 0.52)	0.45 (0.37, 0.53)	0.51 (0.38, 0.60)	0.55 (0.44, 0.68)	0.57 (0.47, 0.68)	0.60 (0.49, 0.73)	0.61 (0.50, 0.74)
G_3_	0.47 (0.38, 0.51)	0.42 (0.37, 0.49)	0.49 (0.40, 0.60)	0.55 (0.45, 0.69)	0.55 (0.46, 0.65)	0.65 (0.50, 0.81)	0.64 (0.50, 0.77)
G_4_	-	-	0.54 (0.48, 0.64)	0.54 (0.42, 0.69)	0.56 (0.46, 0.70)	0.70 (0.54, 0.89)	0.66 (0.52, 0.84)
G_5_	-	-	-	-	0.57 (0.45, 0.73)	0.73 (0.55, 0.94)	0.66 (0.53, 0.80)
G_6_	-	-	-	-	-	-	0.64 (0.54, 0.79)
Threshold							
Mean ± [SD]	0.45 ± [0.12]	0.45 ± [0.11]	0.52 ± [0.18]	0.58 ± [0.19]	0.60 ± [0.20]	0.67 ± [0.24]	0.67 ± [0.23]
Mean + 2SD	0.69	0.67	0.88	0.97	0.99	1.16	1.13
**B**_**wt**_**/A**	**0–3 years**	**3–6 years**	**6–9 years**	**9–12 years**	**12–15 years**	**15–18 years**	**18–24 years**
G_1_	0.16 (0.14, 0.21)	0.16 (0.12, 0.20)	0.15 (0.12, 0.19)	0.13 (0.11, 0.16)	0.13 (0.10, 0.17)	0.14 (0.11, 0.17)	0.12 (0.10, 0.15)
G_2_	0.15 (0.13, 0.18)	0.14 (0.11, 0.16)	0.13 (0.11, 0.15)	0.11 (0.09, 0.14)	0.11 (0.09, 0.14)	0.12 (0.09, 0.15)	0.10 (0.08, 0.13)
G_3_	0.17 (0.14, 0.20)	0.12 (0.10, 0.15)	0.12 (0.10, 0.15)	0.10 (0.08, 0.13)	0.10 (0.08, 0.13)	0.10 (0.08, 0.13)	0.09 (0.07, 0.12)
G_4_	-	-	0.12 (0.10, 0.15)	0.09 (0.07, 0.12)	0.09 (0.07, 0.12)	0.10 (0.08, 0.13)	0.09 (0.07, 0.11)
G_5_	-	-	-	-	0.09 (0.07, 0.11)	0.09 (0.07, 0.12)	0.08 (0.07, 0.10)
G_6_	-	-	-	-	-	-	0.09 (0.07, 0.10)
Threshold							
Mean ± [SD]	0.17 ± [0.07]	0.15 ± [0.05]	0.14 ± [0.05]	0.12 ± [0.05]	0.12 ± [0.05]	0.12 ± [0.05]	0.11 ± [0.04]
Mean + 2SD	0.32	0.26	0.24	0.21	0.22	0.23	0.19
**B**_**wt**_**/B**_**out**_	**0–3 years**	**3–6 years**	**6–9 years**	**9–12 years**	**12–15 years**	**15–18 years**	**18–24 years**
G_1_	0.22 (0.19, 0.25)	0.21 (0.19, 0.23)	0.19 (0.17, 0.22)	0.16 (0.14, 0.18)	0.15 (0.13, 0.18)	0.16 (0.14, 0.18)	0.14 (0.12, 0.16)
G_2_	0.20 (0.18, 0.23)	0.19 (0.16, 0.21)	0.17 (0.15, 0.20)	0.14 (0.12, 0.17)	0.14 (0.12, 0.17)	0.14 (0.12, 0.17)	0.13 (0.11, 0.15)
G_3_	0.20 (0.18, 0.25)	0.18 (0.16, 0.21)	0.16 (0.14, 0.19)	0.13 (0.11, 0.16)	0.13 (0.11, 0.16)	0.12 (0.10, 0.15)	0.11 (0.10, 0.14)
G_4_	-	-	0.15 (0.13, 0.17)	0.12 (0.10, 0.15)	0.12 (0.10, 0.15)	0.11 (0.09, 0.13)	0.11 (0.09, 0.13)
G_5_	-	-	-	-	0.12 (0.10, 0.15)	0.10 (0.08, 0.12)	0.10 (0.08, 0.12)
G_6_	-	-	-	-	-	-	0.10 (0.08, 0.12)
Threshold							
Mean ± [SD]	0.22 ± [0.04]	0.20 ± [0.04]	0.18 ± [0.04]	0.14 ± [0.04]	0.14 ± [0.04]	0.13 ± [0.04]	0.12 ± [0.03]
Mean + 2SD	0.30	0.27	0.26	0.22	0.22	0.22	0.19
**B**_**WA**_**/B**_**OA**_	**0–3 years**	**3–6 years**	**6–9 years**	**9–12 years**	**12–15 years**	**15–18 years**	**18–24 years**
G_1_	0.69 (0.62, 0.75)	0.66 (0.62, 0.71)	0.62 (0.56, 0.69)	0.54 (0.47, 0.60)	0.52 (0.45, 0.59)	0.53 (0.47, 0.59)	0.48 (0.42, 0.54)
G_2_	0.65 (0.60, 0.71)	0.61 (0.55, 0.67)	0.58 (0.50, 0.65)	0.49 (0.42, 0.56)	0.48 (0.42, 0.56)	0.48 (0.41, 0.56)	0.44 (0.39, 0.51)
G_3_	0.65 (0.60, 0.74)	0.60 (0.55, 0.66)	0.55 (0.48, 0.61)	0.46 (0.40, 0.53)	0.46 (0.39, 0.54)	0.42 (0.35, 0.51)	0.40 (0.35, 0.47)
G_4_	-	-	0.52 (0.45, 0.56)	0.43 (0.37, 0.51)	0.43 (0.36, 0.51)	0.38 (0.33, 0.46)	0.38 (0.32, 0.44)
G_5_	-	-	-	-	0.42 (0.35, 0.50)	0.37 (0.30, 0.44)	0.36 (0.31, 0.41)
G_6_	-	-	-	-	-	-	0.36 (0.31, 0.41)
Threshold							
Mean ± [SD]	0.67 ± [0.09]	0.63 ± [0.09]	0.58 ± [0.11]	0.49 ± [0.10]	0.48 ± [0.11]	0.46 ± [0.12]	0.42 ± [0.10]
Mean + 2SD	0.85	0.80	0.80	0.69	0.69	0.69	0.62

The results of segmental generation G_1_ and higher are shown in median (IQR) and the threshold indicate bronchial widening or wall thickening if BA ratios greater than [mean + 2*SD] for all generations by age group. IQR, shown in (25th percentile, 75th percentile); years; G: segmental generations, and G_n+1_: the consecutive branching bronchi; B_out_/A: ratio between bronchial outer wall diameter and adjacent pulmonary artery; B_in_/A: ratio between bronchial inner wall diameter and the adjacent pulmonary artery; B_wt_/A: ratio between bronchial wall thickness and adjacent pulmonary artery diameter; B_wt_/B_out_: ratio between bronchial wall thickness and bronchial outer diameter; B_WA_/B_OA_: ratio between bronchial wall area and bronchial outer wall area

*IQR* interquartile range, *SD* standard deviation

## Discussion

To the best of our knowledge, this is the first large cohort study providing normative CT data encompassing lung density, BA dimensions, and BA ratios in children from preschool age to young adulthood. We have established reference values for several AI-based BA method-derived CT parameters, describing age- and sex-related differences in BA dimensions, BA ratios, lung volume, and density.

Our findings indicate that both bronchial outer and lumen diameters increase with age from above the age of 6 to 24 years. However, the relationship between bronchi and pulmonary artery dimensions demonstrates no age-dependent change aligning with a previous study [11] which showed no correlation in BA ratios with age. This suggests a synchronous growth pattern between bronchi and paired pulmonary arteries. Even though the BA ratios do not change with age, for clinical relevance we propose age-specific upper limits of normal values for defining abnormal bronchial widening and wall thickening (Table 2). Our cutoff values for B_in_/A are similar to the current cutoff values range used for defining bronchial widening in adults, ranging between 1 to 1.5 [2, 13], but they are greater than the range proposed in a study involving children, which ranges between 0.5 and 0.8 [4, 11, 14, 15]. This discrepancy can be explained by different criteria and procedures to define the boundary of the bronchial wall. The automatic BA method is trained using phantoms rather than manual measurements [5]. Our cutoff values for B_out_/A are around 1.5 from 0 to 15 years and larger above the age of 15 to 24 years. We believe that our proposed cutoff values, tailored to patient age and derived from a large dataset, offer enhanced precision compared to those currently in use. These cutoff values can be used to define bronchial pathology in children on both non-contrast and contrast-enhanced CT scans, providing an objective outcome measure that is superior to current radiological visual assessments.

A change in bronchial wall thickness with age was not found. Additionally, regarding all wall thickness-related outcomes (B_wt_/A, B_wt_/B_out_, and B_WA_/B_OA_), bronchial wall thickness became thinner from central to peripheral generations, which is in line with previous results [16]. Pi10 was used to express and evaluate bronchial wall thickening based on a large number of BA measurements over a range of generations [9, 17]. In our study, we noted a decrease in Pi10 for the higher age groups, which implies that the bronchial ratios are a more accurate parameter for evaluating bronchial wall thickening than Pi10 as it is independent of age and size-dependent changes in bronchial diameters.

Interestingly, we observed a slight increase in mean lung density from age above 6 to 24 years, which contrasts with the findings of a previous study. Another study showed that the mean lung density decreases linearly from birth to adulthood when adjusted by age and height [18]. We speculate that this discrepancy might be due to our inclusion of more confounders (age, sex, total lung volume, and contrast conditions) in our models. Mean lung density is influenced by the relative amount of air and tissue volume within a given voxel [19], and as such, changes in mean lung density are likely affected by the degree of lung inflation. As our chest CT scans were not spirometry-controlled, the discrepancy could be explained by varying inspiratory levels achieved by the study subjects following the technicians’ commands. To mitigate this effect, we visually checked the lung inflation, with 96% of CT scans deemed to have moderate to good lung inflation. While further studies with standardization of CT volume are essential to elucidate the shifts in mean lung density during growth, our results suggest that the current cutoff values for air trapping (< −856 HU) [20, 21] and emphysema (−950 HU) [22] used in adult thoracic imaging may need to be adjusted for children.

We observed sex differences in both total lung volume and bronchial outer diameter. While the former aligns with existing literature [23], we cannot dismiss the possibility that the observed sex differences in bronchial outer diameter might be a type 1 error, especially considering that bronchial lumen and wall thickness remained consistent across sexes. These findings match those of previous studies on central airways [6] challenging the long-standing assumption that females have inherently smaller airways [24]. Our results indicate that the difference in bronchial size between sexes primarily lies in the bronchial outer diameter, a distinction observed consistently from youth to middle age [25].

In our study, participants younger than 6 years were excluded from statistical analysis for two main reasons. First, despite the moderate to good lung inflation of the included CT scans, most participants aged 6 years or younger were typically unable to perform a voluntary breath-hold scan. Hence scans are typically obtained at lower lung volumes, within the functional residual capacity range, rather than close to total lung capacity. Suboptimal inspiration during CT scans results in reduced dimensions, especially of the bronchial lumen diameter [26–28], and an increase in wall thickness. Reduced lumen diameter may lead to an underestimation of bronchial widening, while bronchial wall thickness may be overestimated, as illustrated in Fig. 5, which presents boxplots for participants younger than 7 years. Second, the CT scan parameters used for younger participants are often different compared to those used in older participants, which are routinely set at a lower radiation dose for safety concerns (Table S1a, S1b). This lower radiation dose could potentially result in reduced image quality and sharpness, limiting the software’s ability to accurately measure bronchial wall dimensions. As a result, we excluded these younger participants from the analysis.

The overall agreement between the two observers was fair, which could be attributed to the different aims of the studies for which the data have been used. The first observer who selected the CT scans for the previous study focused on central airway assessment and parenchymal abnormalities, while the second observer for the current study selected scans focusing on peripheral airways and lung density. CT scans excluded from the previous study analysis included abnormalities, such as bronchial wall thickness, small nodules, or cysts. Abnormalities detected by one or both observers were excluded.

### Limitations

There are some limitations to our study. First, its retrospective nature resulted in a wide range of CT protocols being used and suboptimal volume optimization representing current practices across multiple centers. Nevertheless, we would like to emphasize that these real-world data provided valuable insight into the developmental trends of bronchial dimensions with growth, aiding in the definition of bronchial widening and wall thickening in children. While the retrospective design inherently limits control over data collection and introduces the potential for selection bias (Appendix S3), we believe that including data from multiple international centers with diverse patient populations and varying CT protocols strengthens the study’s generalizability to a wider range of populations.

Second, we were unable to adjust our study results for known confounders in respiratory function studies, such as height, or body mass index, as these data were not available in relation to consent restrictions. Most importantly, the subject’s height is directly related to airways size and total lung volume. To reduce the impact of this missing variable, we corrected the airways measurements for age, sex, and total lung volume as obtained from the CT data. We believe that this correction is likely to have largely corrected the potential effect of length on bronchial anatomy and development.

Finally, it is important to acknowledge that the CT dataset included patients who underwent CT for various clinical reasons. Therefore, we could not entirely exclude the presence of subtle lung pathology in these participants. To mitigate this problem, normal CT scans were assessed by two independently trained radiologists to detect any lung abnormality. Despite these limitations, this dataset is currently the most suitable and practically the best available to generate reference values to provide guidance for the evaluation of chest CT scans in children from preschool age to young adulthood. Our findings highlight the critical influence of a CT protocol and volume standardization, emphasizing the ongoing need for the continued collection of normal chest CT scans. This effort is crucial for further refinement and optimization of reference values, ultimately advancing the reliability and clinical utility of these diagnostic tools.

## Conclusions

Our study provides comprehensive and valuable normative CT data for BA dimensions, BA ratios and lung density from childhood into young adulthood without the use of volume standardization. We have successfully established novel age-dependent reference values for several AI-based BA method-derived CT parameters, showing age and sex-related differences in bronchial dimensions, lung volume and density. We observed a consistent age-related synchronous growth pattern between bronchi and paired pulmonary arteries. We propose age-specific upper limits of normal values to define abnormal bronchial widening or wall thickening, offering enhanced precision compared to current standards. These cutoff values have the potential to aid in the diagnosis of bronchial disease in pediatric and young adult populations. Further studies are needed to collect chest CT scans with volumetric control and incorporate additional respiratory-related parameters (such as height, weight, and body mass index) to accurately investigate bronchial development. The proposed cutoff values could facilitate the automatic detection and quantification of bronchial disease, as well as the assessment of treatment effects.

## Supplementary information

ELECTRONIC SUPPLEMENTARY MATERIAL

## Abbreviations

A: Adjacent pulmonary artery diameter
AI: Artificial intelligence
BA: Bronchus-artery
B_in_: Bronchial lumen diameter
B_in_/A: The ratio of bronchial lumen diameter and adjacent artery diameter
B_out_: Bronchial outer diameter
B_out_/A: The ratio of bronchial outer diameter and adjacent artery diameter
B_WA_/B_OA_: The ratio between bronchial wall area and bronchial outer wall area
B_wt_: Bronchial wall thickness
B_wt_/A: The ratio of bronchial wall thickness and adjacent artery diameter
B_wt_/B_out_: The ratio between bronchial wall thickness and bronchial outer diameter
G: Segmental bronchial generation
Pi10: The square root of wall area of a 10-mm lumen perimeter
