# Supplementary material for: Normative values for lung, bronchial sizes, and bronchus-artery ratios in chest CT scans: from infancy into young adulthood
Source: Eur Radiol. 2025 Feb 1;35(8):4846–60. doi: 10.1007/s00330-025-11367-w (PMC12226683; doi:10.1007/s00330-025-11367-w)
Supplement: Supplementary file 1 — ELECTRONIC SUPPLEMENTARY MATERIAL [file 330_2025_11367_MOESM1_ESM.pdf]

Normative values for lung, bronchial sizes and bronchus-artery ratios in non-contrast and contrast-enhanced chest CT scans: a study of 375 patients from infancy into young adulthood

ELECTRONIC SUPPLEMENTARY MATERIAL

Table of contents

S1 Participating Centers

S2 Standard Operation Procedure (Image Quality and Volume Quality Assessment)

S3 The potential sources of bias for the analysis

Supplementary Figure

Figure S1. Distribution of BA-pair numbers in CT scans across age groups

Supplementary Tables

Table S1a. Summary of CT scanning parameters in each age group

Table S1b. Summary of CT scanning parameters of included scans and excluded scans

Table S2. Median and IQR of bronchial dimensions in non-iodine contrast (n=210) CT scans, categorized by segmental generation and age group

Table S3. Influence of age, sex, total lung volume, and iodine contrast on bronchial dimensions (mm)

Table S4. Influence of age, sex, total lung volume, and iodine contrast on BA-ratios

Table S5. Median and IQR of total lung volume, mean lung density, and Pi10 according to age group in non- iodine contrast scans

Table S6. Effect of age, sex, mean lung density(HU), iodine contrast, and CT scanners on total lung volume(mL)

S1: Participating Centers

The following centers contributed to the Normal Chest CT Study Group dataset (ordered according to number of CT scans provided, from highest to lowest):

University Medical Center Utrecht, Utrecht, The Netherlands;

Hospital Universitari Vall d'Hebron, Barcelona, Spain;

Erasmus University Medical Center, Sophia Children's Hospital, Rotterdam, The Netherlands;

University of Rome Sapienza, Rome, Italy;

Cincinnati Children's Hospital Medical Center, Cincinnati, OH;

Boston Children's Hospital and Harvard Medical School, Boston, MA;

Queen Silvia Children's Hospital, University of Gothenburg, Gothenburg, Sweden;

University of Leuven, Leuven, Belgium;

General Hospital, Ospedale Ca' Foncello, Treviso, Italy;

Princess Margaret Hospital for Children and Telethon Kids Institute, Perth, Western Australia.

S2 Standard Operation Procedure (Image Quality and Volume Quality Assessment)

[redacted]	Image Quality and Volume Quality Assessment	S096 – V1.0 / 24-02-2021
[redacted]	STANDARD OPERATING PROCEDURE	Page **1** of **15**

SOP S096: Image Quality and Volume Quality Assessment

Standard Operation Procedure Document of
[redacted]

Document Details:

**SOP number:**	S096
**SOP name:**	Image Quality and Volume Quality Assessment
**SOP hierarchy:**	Procedure SOP
**Initiator(s):**	[redacted]
**Author(s):**	[redacted]<br>☐ ____________
**Reviewer(s)**	[redacted]<br>☐ ____________

1. PURPOSE

The purpose of this standard operating procedure (SOP) is to provide a clear procedure for researchers who have to perform an image quality and/or volume quality assessment.

2. SCOPE

This SOP ensures that image quality and volume quality assessment is done by all observers in a standardized manner.

3. DEFINITIONS AND ABBREVIATIONS

IQ	Image Quality
VQ	Volume Quality
CT	Computed Tomography
TLC	Total Lung Capacity
RV	Residual Volume
TV	Tidal Volume
FRC	Functional Residual Capacity

4. RESPONSIBILITIES

4.1[redacted]

[redacted] responsible for distributing this SOP to the appropriate staff and keeping a copy of the current SOP.

4.2. Site staff

The Site staff, e.g. PhD students, master students, scoring personnel, research technician] are responsible for reading, understanding, and following the current SOP

5. PROCEDURES

5.1. Image Quality Assessment

There are three levels of image quality (IQ) status ascribed to CT scans: Good, Moderate and Bad. In general, CTs classified with a good or moderate IQ are suitable for PRAGMA-CF image analysis. Some problems could be expected with the automatic image analysis tools.

The images classified as 'bad' are more difficult to analyze using the PRAGMA-CF image analysis method. Automatic software tools will most likely not be successful in analyzing these images. All CTs classified as 'bad' will be assessed per case if they will be excluded for PRAGMA-CF or automatic image analysis software tools or not.

5.1.1. Image quality level: good

No impediments for visual assessment and (semi) quantitative scoring. No parts of the lung parenchyma are obscured. Anatomical structures and pathological features can clearly be identified.

The following criteria apply:

- None or minimal motion artifacts of the patient
- None or minimal motion artifacts of diaphragm
- None or minimal motion artifacts of the heart

- None or minimal CT reconstruction artifacts (e.g. streaking, beam hardening)
- None or minimal hardware-based artifacts (e.g. ring artifact)

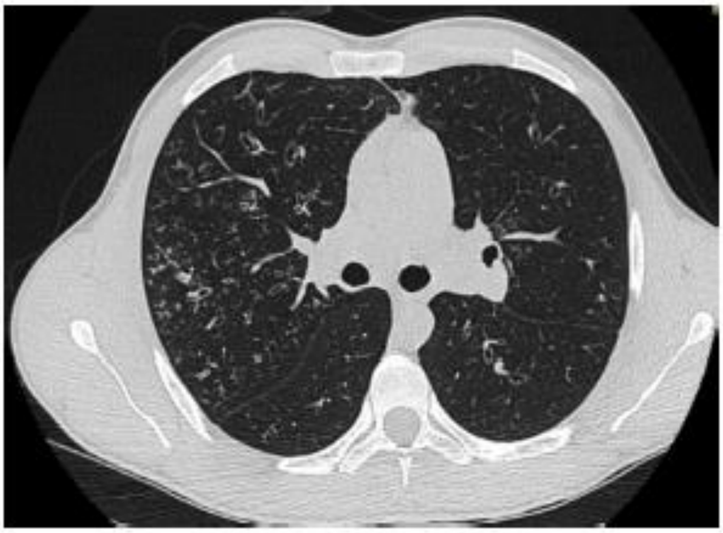

Example (1): Good image quality. No distortion and no parts of the parenchyma are obscured. Anatomical structures and pathological features can readily be identified.

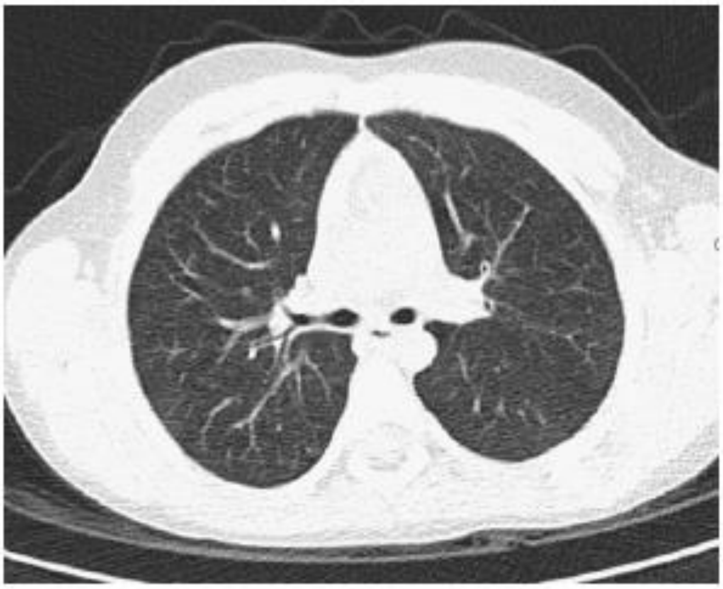

Example (2): Good image quality. No distortion and no parts of the parenchyma are obscured. Anatomical structures and pathological features can readily be identified.

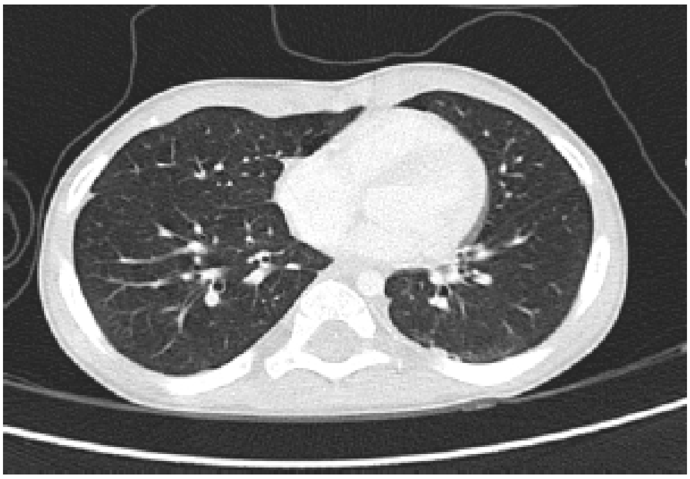

Example (3). Good image quality. Minimal cardiac motion artifacts, causing minimal distortion of the lung parenchyma around the heart.

5.1.2. Image quality level: moderate

Minor impediments for visual assessment and (semi) quantitative scoring. Small parts of the lung parenchyma are obscured. Anatomical structures and pathological features can be identified, albeit with some difficulty.

At least one of the following criteria applies:

- Minor motion artifacts of the patient
- Minor motion artifacts of diaphragm
- Minor motion artifacts of the heart
- Minor CT reconstruction artifacts (e.g. streaking, beam hardening)
- Minor hardware-based artifacts (e.g. ring artifact)

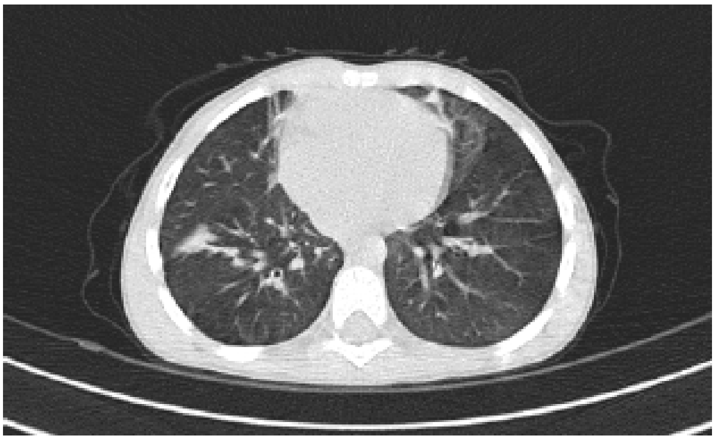

Example (1): Moderate image quality. There is some distortion and obscured parenchyma in both lungs, but anatomical structures and pathological features can be identified.

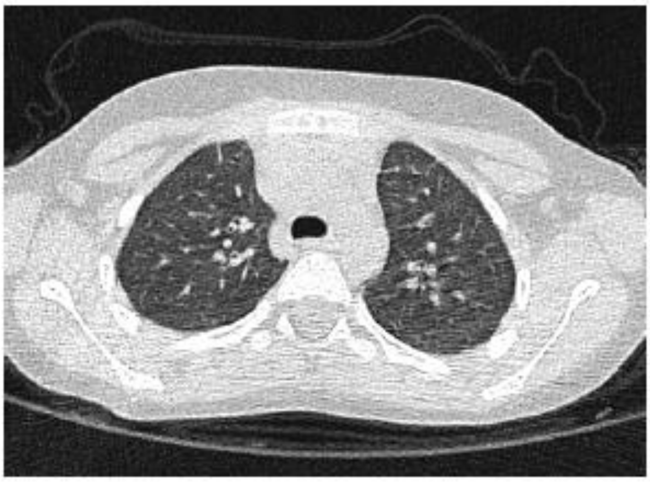

Example (2): Moderate image quality. There is some distortion and obscured parenchyma in the left lung, but anatomical structures and pathological features can be identified.

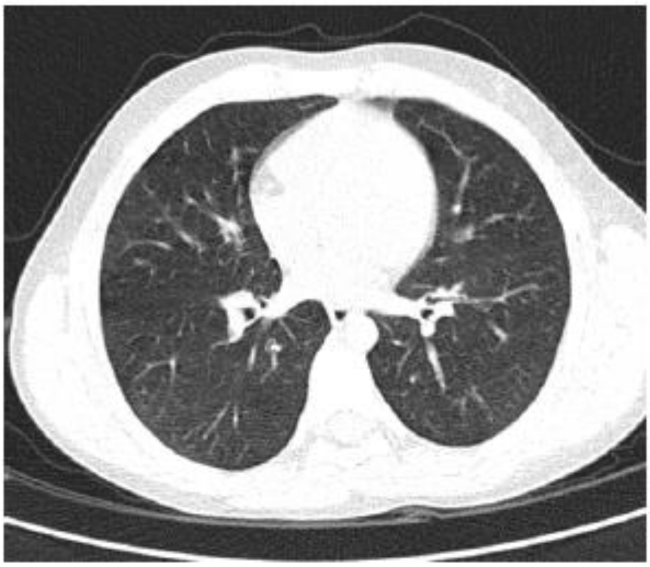

Example (3): Moderate image quality. Cardiac motion artifacts causing some distortion of the parenchyma around the heart in both lungs, but not enough to prohibit visual assessment or (semi) quantitative scoring.

5.1.3. Image quality level: bad

Major impediments for visual assessment and (semi) quantitative scoring. Large parts of the lung parenchyma are obscured. Anatomical structures and pathological features cannot be readily identified.

At least one of the following criteria applies:

- Major motion artifacts of the patient
- Major motion artifacts of diaphragm
- Major motion artifacts of the heart
- Major reconstruction artifacts (e.g. streaking, beam hardening)
- Major hardware-based artifacts (e.g. ring artifact)

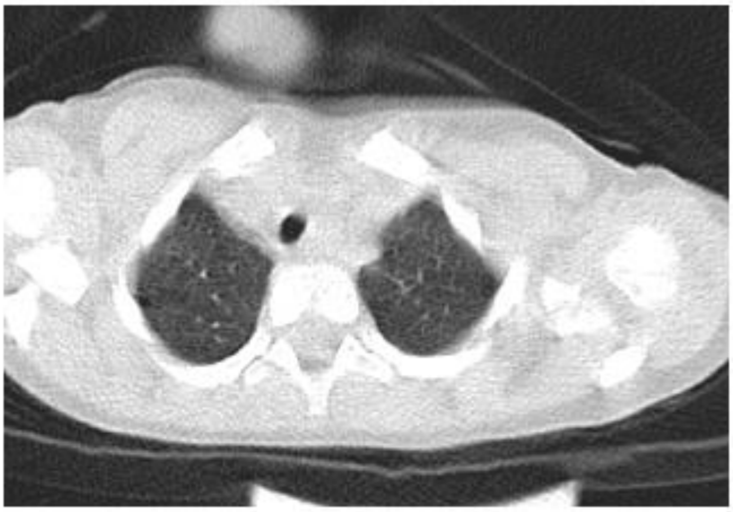

Example (1): bad image quality. Both lungs are obscured, prohibiting visual assessment and (semi) quantitative scoring.

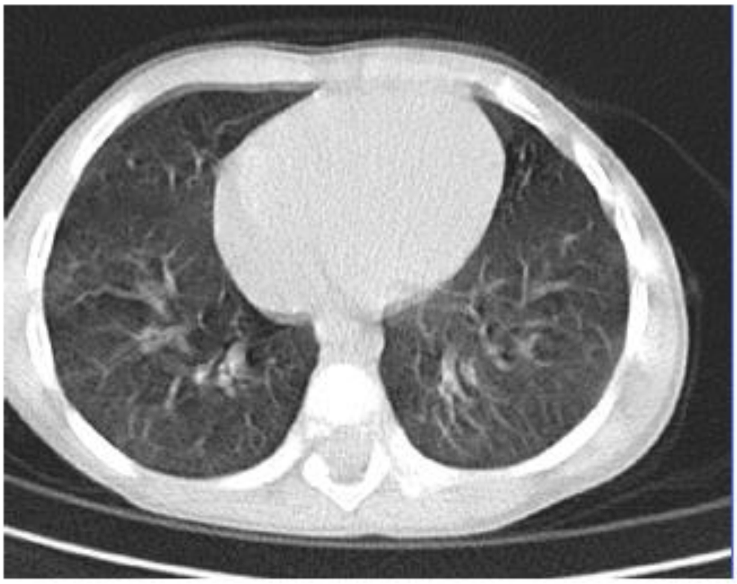

Example (2): bad image quality. Severe distortion in both lungs prohibit visual assessment and (semi) quantitative scoring.

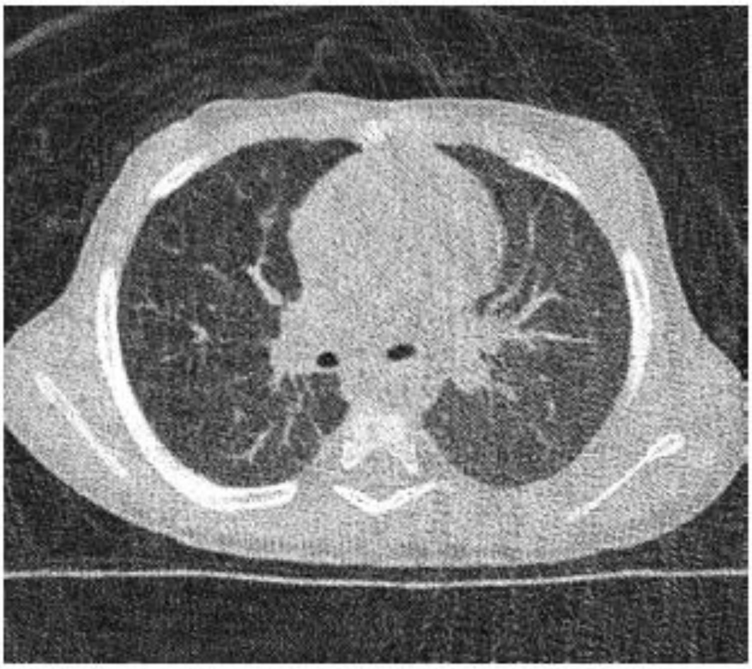

Example (3): bad image quality due to hardware-based artifacts. Visual assessment and (semi) quantitative scoring is prohibited.

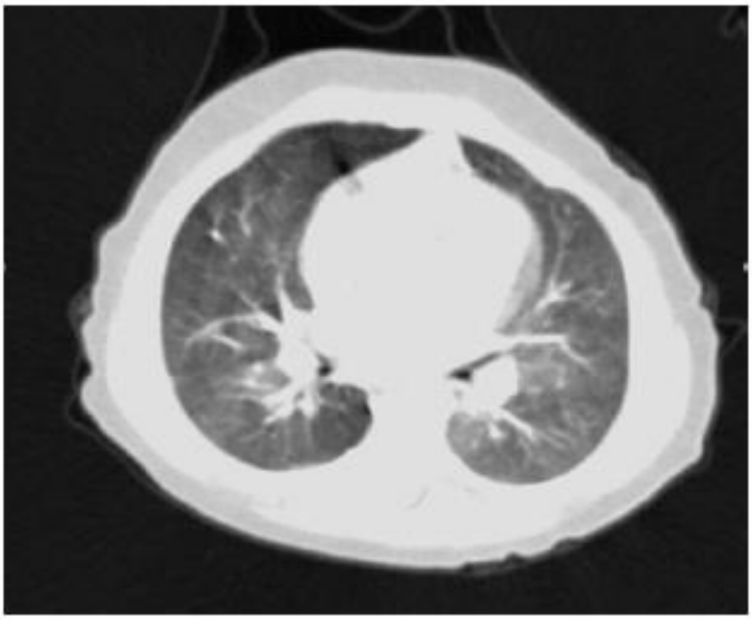

Example (4): bad image quality due to severe cardiac motion artifacts obscuring large parts of the parenchyma.

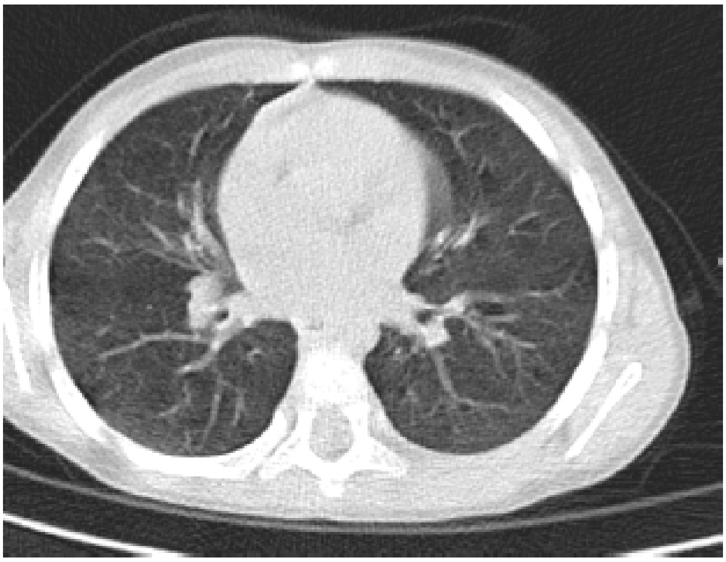

Example (5): bad image quality due to severe cardiac motion artifacts obscuring large parts of the parenchyma.

5.2. Volume Quality Assessment

There are three levels of volume quality (VQ) status ascribed to CT scans: Good, Moderate and Bad. These levels reflect the patient's effort to inhale or exhale from a tidal breathing pattern.

(!) Please note that scrolling through a CT scan is recommended for the proper assessment of volume quality.

The criteria for each of the abovementioned levels are listed below.

5.2.1. Volume quality level: good

Good effort by the patient to inhale or exhale from a tidal breathing pattern, resulting in optimal lung volumes for visual assessment and (semi) quantitative scoring.

The following criteria apply:

- Inspiratory scan with a lung volume at or near total lung capacity (TLC)
- Expiratory scan with a lung volume at or near residual volume (RV)

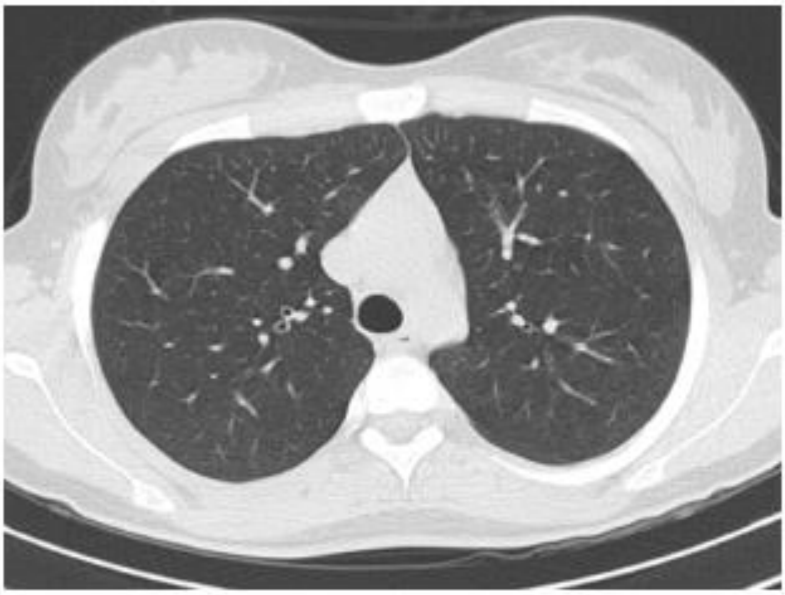

Example (1): Good inspiratory volume quality. Note the posterior tracheal wall is convex-shaped, reflecting good inspiratory effort (i.e. oval-shaped tracheal cross section). The mediastinal compartment lies free from the thoracic wall.

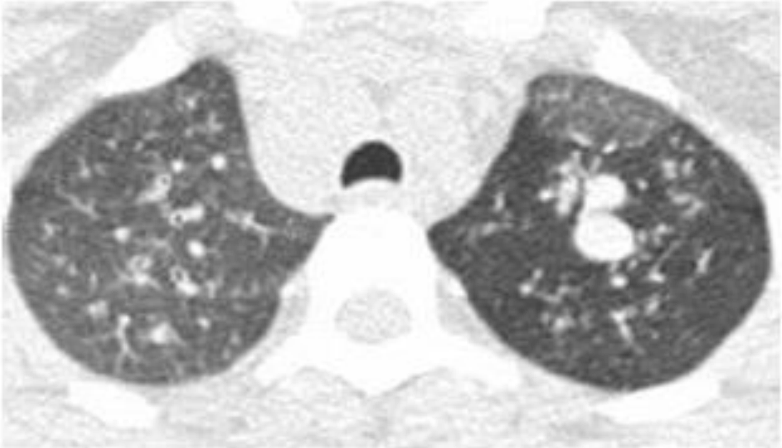

Example (2): Good expiratory volume quality. Note the concavity of the posterior wall of the trachea (arrow-shaped tracheal cross section). Also note the increased parenchymal density showing major differences in attenuation due to trapped air and/or hypo perfusion. The mediastinal compartment touches the thoracic wall.

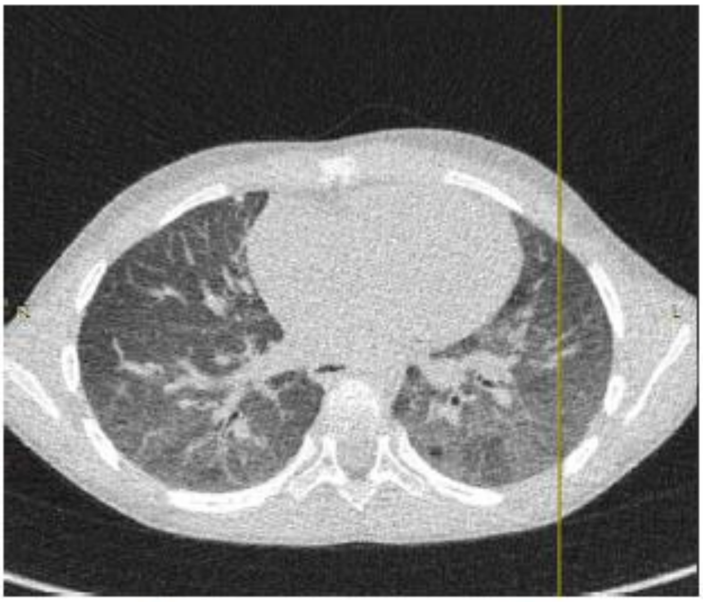

Example (3): Good expiratory volume quality. Note the position of the heart touching the thoracic wall. Also note the increased density of the parenchyma and the flattened right main stem bronchus due to maximum expiratory effort.

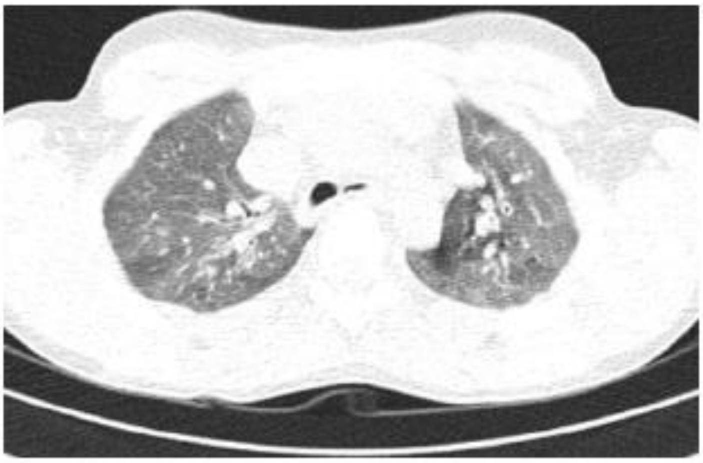

Example (4): Good expiratory volume quality. Note attenuation differences in both lungs allow for reliable differentiation between healthy parenchyma and abnormal parenchyma (trapped air).

5.2.2. Volume quality level: moderate

Moderate effort by the patient to inhale or exhale from a tidal breathing pattern, resulting in sub-optimal lung volumes for visual assessment and (semi) quantitative scoring.

The following criteria apply:

- Inspiratory lung volume less than TLC, but more than FRC+TV
- Expiratory lung volume less than FRC, but more than RV.

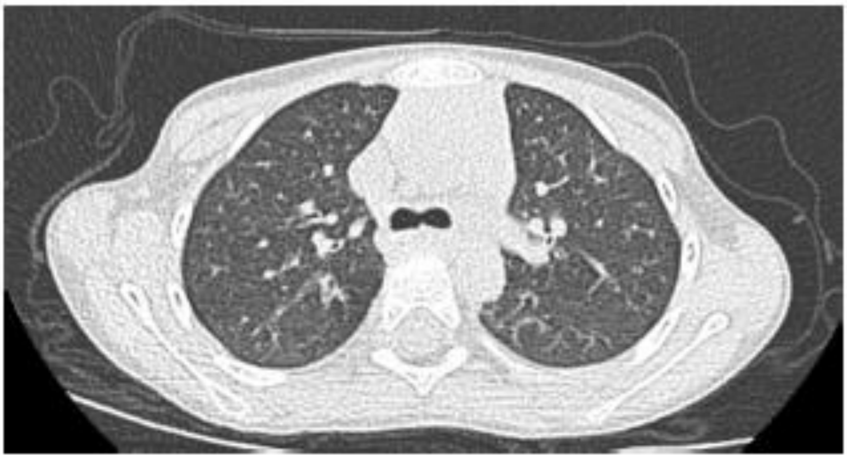

Example (1): Moderate inspiratory volume quality. Note the sub-optimal inflation of the lungs due to subpar inspiratory effort. Also note the undulating lung borders, reflecting a level of inflation below TLC.

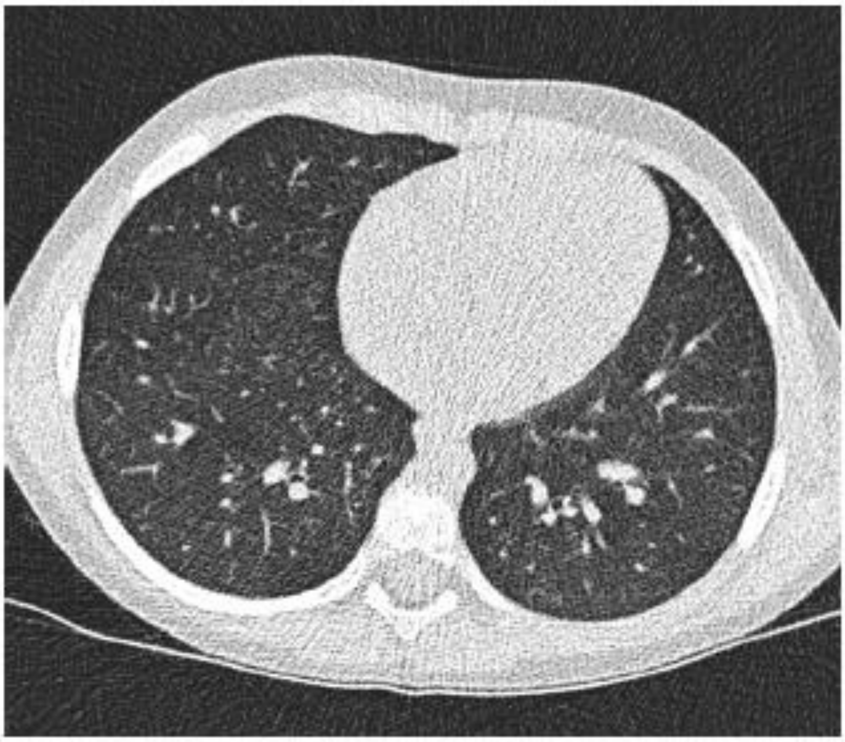

Example (2): Moderate inspiratory volume quality. Again note the undulating lung borders, reflecting a level of inflation below TLC.

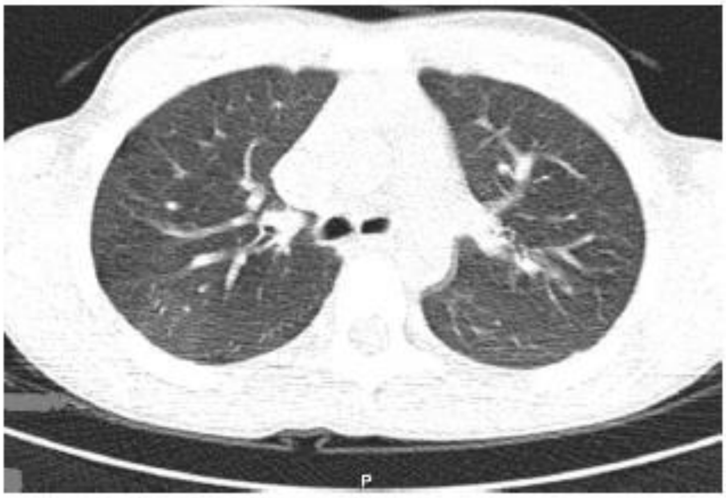

Example (3): Moderate expiratory volume quality due to sub-optimal expiratory effort. Note attenuation differences in both lungs are not pronounced enough to reliably differentiate healthy parenchyma from abnormal parenchyma (trapped air).

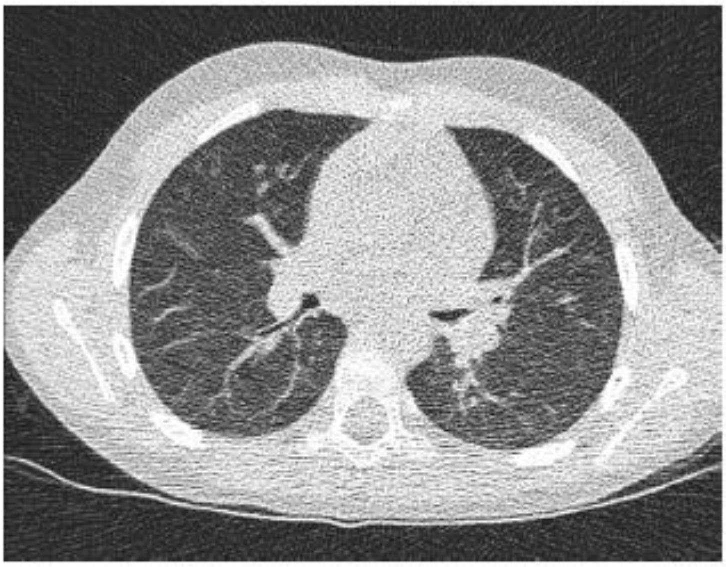

Example (4): Moderate expiratory volume quality. Again note attenuation differences in both lungs are not pronounced enough to reliably differentiate healthy parenchyma from abnormal parenchyma (trapped air).

5.2.3. Volume quality level: bad

Bad effort by the patient to inhale or exhale from a tidal breathing pattern or a volume level opposite that of the reference level (i.e. inspiratory resp. expiratory scan sequence executed at expiratory resp. inspiratory lung volumes).

The following criteria apply:

- Inspiratory lungvolume below FRC+TV
- Expiratory lungvolume above FRC

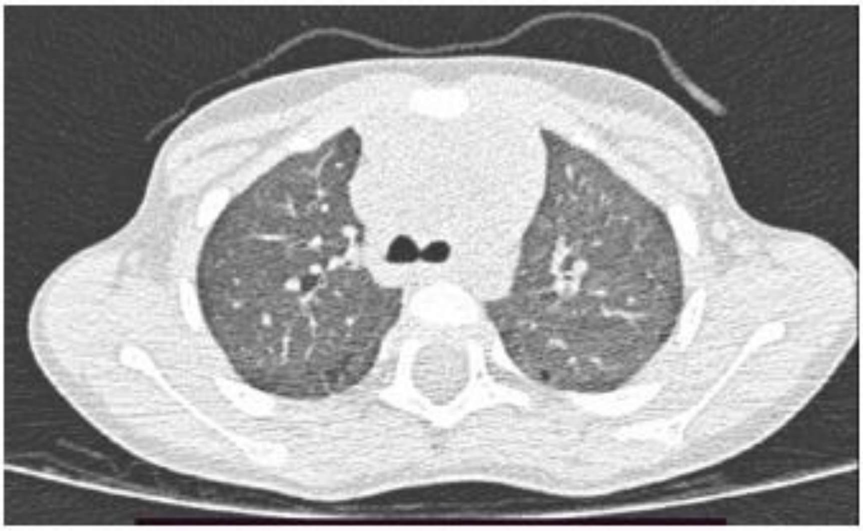

Example (1): Bad inspiratory volume quality. Note the volume level is below FRC. Also note the increased parenchymal density.

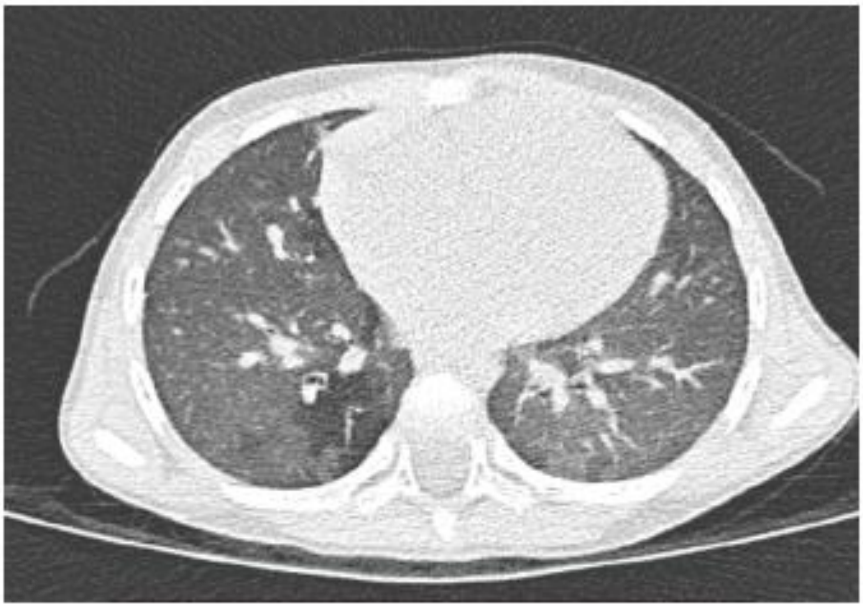

Example (2): Bad inspiratory volume quality. Note the volume level is below FRC and the mediastinal compartment is bordered against the thoracic wall due to the deflated status of the lungs.

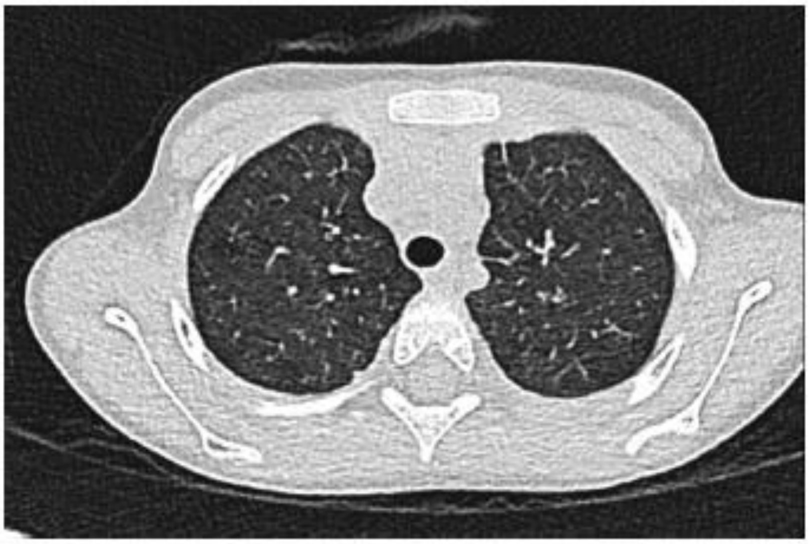

Example (3): Bad expiratory volume quality. Note the convex (oval) shape of the trachea and a volume level above FRC.

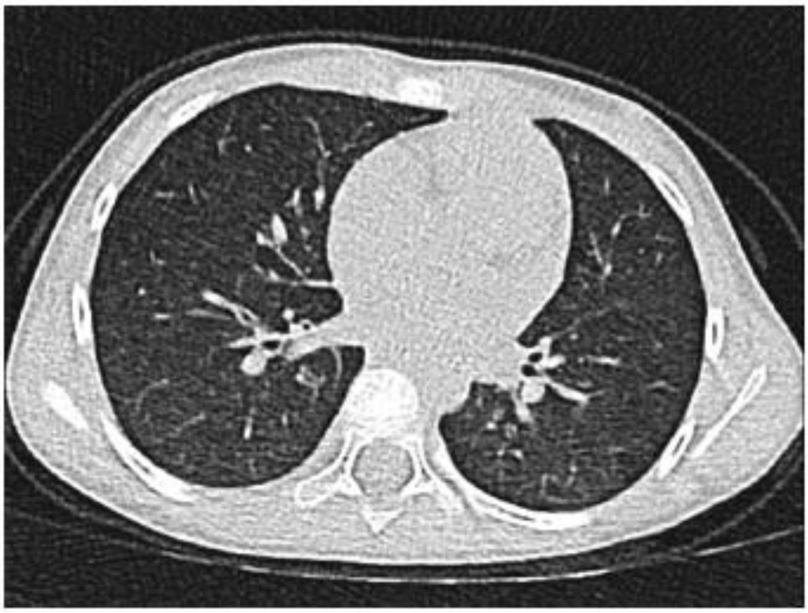

Example (4): Bad expiratory volume quality. Note the low attenuation of the parenchyma and volume level above FRC+TV.

6. Equipment / qualifications

Computer 10); high resolution monitor; DICOM viewing software (e.g. RadiAnt).

[redacted]

CT images that are analysed [redacted]nd all accompanying digital documents will be stored within the [redacted]rastucture. All research data will be saved for 15 years after completion of the project. Long term archiving will be done on archiving Servers [redacted]

8. Document revision history

[Version / date]	[reason for change]
1.0 / 24-02-2021	Original document

9. Appendix I: Lung Volumes

Please see next page.

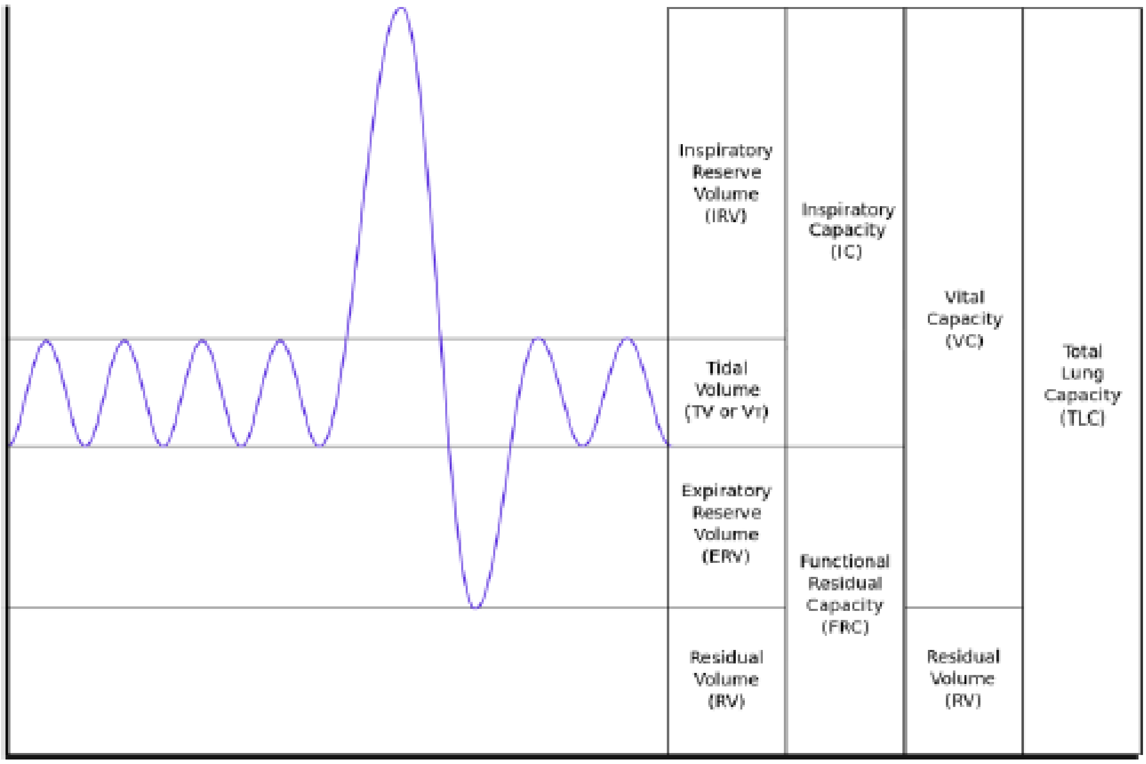

S3 The potential sources of bias for the analysis

In this analysis of airway dimensions from chest CT data of children, there are several potential sources of bias that could influence both the direction and magnitude of the findings. These biases could arise from the dataset itself, the study design, and the statistical model used:

1.Selection Bias

- **Direction**: Selection bias might occur if the cohort is not fully representative of the general pediatric population. Since CT scans were deemed "normal" by three radiologists, there is still a possibility that the sample includes children with subtle, undetected abnormalities, or conversely, excludes some normal variations in airways. This could skew the results toward an idealized population, possibly underestimating natural variability.
- **Magnitude**: The magnitude of this bias may be moderate, as selection by radiologists introduces a subjective element. While experienced radiologists might have high agreement on what constitutes a normal CT, small differences in interpretation can influence the cohort composition.

2. Measurement Bias

- **Direction**: Measurement bias may arise from inconsistencies in CT scanner types (Philips, SIEMENS, TOSHIBA) and the use of iodine contrast. Although these are included as fixed effects in the model, differences in scanner resolution could still introduce variability in the measurements of airway dimensions.
- **Magnitude**: The bias should be small. While the mixed-effects model accounts for scanner type and contrast use, these factors might still affect the absolute airway measurements due to subtle differences in imaging quality or protocol.

3.Observer Bias

- **Direction**: Although the model uses objective airway measurements, human intervention in data preparation, such as determining airway generations or identifying key anatomical landmarks, might introduce observer bias. This could skew results if certain airways or measurements are systematically under-estimated by software algorithms or spatial resolution (i.e. airways generation smaller than 6th generation).
- **Magnitude**: The magnitude of observer bias may be small, especially if the process of identifying and measuring airways is standardized, as in our study by using an AI algorithm.

4.Confounding Bias

- **Direction**: Potential confounders, such as unmeasured environmental factors (e.g., exposure to pollutants or allergens), genetic variations, indication to perform the CT, are not included in the model. These factors could influence airway dimensions and introduce bias in the relationships observed between the fixed effects (age, sex, total lung volume) and airway measurements.
- **Magnitude**: The confounding bias could be moderate to large, depending on the impact of these unmeasured variables on airway dimensions. For instance, regional or genetic factors could significantly influence bronchial anatomy, leading to biased

estimates of the true population-level relationships.

5.Modeling Bias

- **Direction**: The use of a mixed-effects model helps address the hierarchical nature of the data (multiple measurements per subject, multiple airway generations), but the assumptions inherent in the model could still introduce bias. For example, treating participants, lobes, and segmental generations as random effects assumes that variations within these groups are normally distributed. If the true variability is non-normal, this could bias the model's estimates.
- **Magnitude**: The magnitude of modeling bias is likely small, as mixed-effects models are robust for dealing with repeated measures and hierarchically structured data.

6.Residual Confounding and Interaction Effects

- **Direction**: If there are unaccounted interaction effects between variables (e.g., between age and sex, or lung volume and airway generation), the model could miss important nuances, potentially leading to biased or incomplete interpretations of the relationships between the measured variables.
- **Magnitude**: The magnitude of this bias could be moderate if significant interactions are overlooked. For example, airway growth patterns might differ based on both sex and age, and without modeling these interactions, the fixed effects may not fully capture the complexity of airway development.

Overall Impact on Findings

The direction of potential biases could lead to both over- and under-estimations of airway dimensions and ratios, depending on how each source of bias influences the measurements. The magnitude of bias is likely to be small to moderate overall, given the use of a robust mixed-effects model, but factors like unmeasured confounders or subtle differences in scanner performance could still affect the precision of the estimates. These biases may affect the generalizability of the findings to broader pediatric populations and could either exaggerate or underestimate the true variability in airway dimensions.

To mitigate these biases we mentioned in the conclusions, that it is essential to validate the results in a larger and more diverse cohorts, improve standardization of imaging protocols, including lung volume standardization, and consider additional confounders in future analyses. Due to the words count limit, we have added this consideration in the online supplement.

Supplementary Figure

Figure S1. The number of BA-pairs per segmental generation in each age group

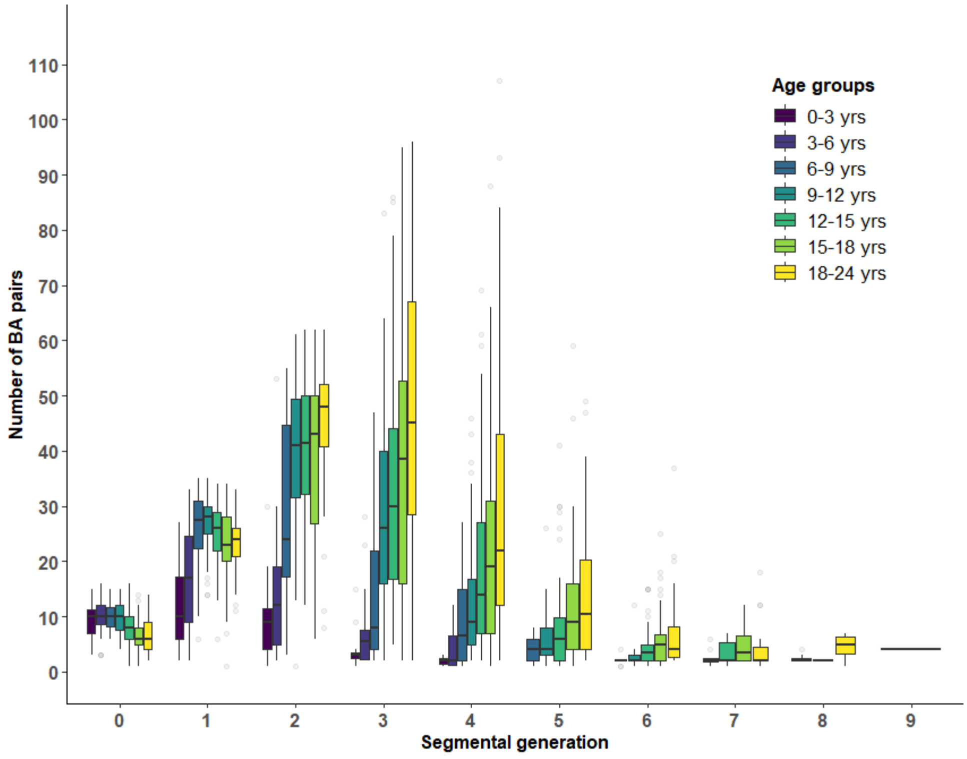

BA-pair: bronchus-artery pair; CT: computed tomography. Boxplot of the BA-pair number compared to the segmental generations. In the horizontal axis of figure S1, segmental generation 0 indicates the segmental bronchi and 1 indicates consecutive sub-segmental bronchi and so forth. The number of BA-pairs represent the number of detectable bronchial branches. Note that according participant's group, bronchi located more distally from segmental generation 4 are present in lower numbers or not visible especially in the younger age groups due to the resolution of the CT scanner. Some adjacent arteries originating from G_0 were challenging to detect due to their clustered anatomical structure. Thus unpaired bronchial measurements (lacking paired arteries) were excluded from the dataset. As a result, the number of branching artery pairs from G_0 ranged between 7 to 10.

Supplementary Tables

Table S1a. Summary of CT scanning parameters in each age group

Age groups	0-3 yrs	3-6yrs	6-9yrs	9-12yrs	12-15 yrs	15-18 yrs	18-24 yrs
**Parameter**							
**No. of scans (with contrast)**	24 (6)	23 (5)	34 (10)	71 (29)	82 (28)	85 (48)	56 (39)
**Age at scanning (years)- median (IQR)**	1.6 (1.4, 2.3)	4.8 (3.8, 5.3)	7.7 (6.7, 8.3)	10.9 (10.0, 11.4)	13.9 (12.8, 14.5)	16.8 (15.8, 17.4)	19.0 (18.5, 19.6)
**Sex**							
Male	15	16	13	44	56	42	33
Female	9	7	21	27	26	43	23
**CT scanners**							
GE Healthcare						1	
Philips Healthcare	8	15	16	47	44	54	37
Siemens Healthcare	13	7	12	21	32	22	18
Toshiba Medical Systems	3	1	6	3	6	8	1
**Reconstruction kernel**							

GE Healthcare						STANDARD	
Philips Healthcare	D, L, YA	YA	C, L, YA	B, C, L, YA, YB	B, C, L YA, YB	B, C, L, YA, YB, YC	B, C, L, YA
Siemens Healthcare	B60f,<br>B70f,<br>B70s,<br>B75f	B30f,<br>B70f,<br>B70s,<br>B75f	B50f,<br>B60f,<br>B70f,<br>B75f	B31f,<br>B60f,<br>B60s,<br>B70f,<br>B70s,<br>B75f	B46f,<br>B50f,<br>B60f,<br>B60s,<br>B70f,<br>B70s,<br>B75f	B26f,<br>B26s,<br>B30f,<br>B60f,<br>B70f,<br>B70s,<br>B75f	B30f,<br>B50f,<br>B60f,<br>B60s,<br>B70f
Toshiba Medical Systems	FC12, FC18	FC18	FC12, FC18	FC12, FC18	FC12, FC18	FC12, FC18	FC18
**Tube voltage (kV)**	100 (80-120)	120 (105-120)	100 (90-120)	110 (90-120)	100 (90-120)	120 (120-120)	120 (120-120)
**Pitch**	2.2 (1.5-3.0)	1.9 (0.9-3.0)	0.8 (0.8-1.3)	0.8 (0.8-1.1)	0.8 (0.8-0.8)	0.8 (0.6-0.9)	1.0 (0.9-1.2)
**Slice thickness (mm)**	1 (0.75-1.50)	1.5 (1.12-1.50)	1 (0.75-1.00)	1 (1.00-1.50)	1 (1.00-1.25)	1 (0.9-1.00)	1 (0.9-1.31)
**Current-time product (mAs)**	65<br>(44.95, 190.95)	66.41<br>(45.79, 91.10)	105<br>(60, 148.93)	105.28<br>(75, 166.83)	137.37<br>(101.15, 223.76)	201.93<br>(114.57, 303.28)	228<br>(168.48, 302.38)
**CT dose index (mGy)**	1.00<br>(0.57, 1.63)	1.06<br>(0.88, 1.62)	1.27<br>(1.06, 2.79)	2.05<br>(1.30, 2.96)	2.30<br>(1.70, 3.72)	5.40<br>(3.05, 10.10)	7.79<br>(5.22, 10.10)

The parameters are shown with median, interquartile range (Q1, Q3).

Table S1b. Summary of CT scanning parameters of included scans and excluded scans

**Parameter**	**Included Scans**	**Excluded Scans**[a]	***P***
**No. of scans**	375	40	
**Age at scanning (years)**	13.31 (9.86, 16.77)	3.38(1.60, 11.33)	<0.001[b]
**Sex**			0.74
Male	219	23	
Female	156	17	
**Tube voltage (kV)**	120 (90-120)	120 (80-120)	0.17
**Pitch**	1.2 (0.85, 1.50)	1.4 (0.84-2.15)	0.38
**Slice thickness (mm)**	1 (0.90, 1.50)	1 (0.75, 1.50)	0.90
**Current-timeproduct (mAs)**	146.64(88.84,238)	134.35(83.46,230.29)	0.13
**CT dose index (mGy)**	2.51 (1.30, 5.40)	2.70 (1.38, 5.40)	0.05

Note—Values are median with interquartile range in parentheses. The T-test was performed to assess difference between the scans included and excluded. [a] Failed to analyzed by automatic BA-method and detected BA-pairs less than 10.[b] This difference mainly due to excluded scans are from younger participants (aged 0 to 6 years).

Table S2. Median and IQR of bronchial dimensions in non-iodine contrast (n=210) CT scans, categorized by segmental generation and age group

B_{out}	0-3Y<br>n= 18	3-6Y<br>n= 18	6-9Y<br>n= 24	9-12Y<br>n= 42	12-15Y<br>n= 54	15-18Y<br>n= 37	18-24Y<br>n= 17
G_1	3.41 (3.06, 3.95)	3.44 (3.08, 3.92)	3.52 (3.10, 4.05)	3.73 (3.12, 4.42)	3.96 (3.23, 4.68)	4.11 (3.40, 4.85)	4.43 (3.75, 5.11)
G_2	3.17 (2.77, 3.54)	2.95 (2.58, 3.44)	2.96 (2.61, 3.34)	2.98 (2.60, 3.48)	3.07 (2.65, 3.65)	3.18 (2.70, 3.85)	3.34 (2.83, 3.98)
G_3	2.69(2.38, 3.08)	2.73 (2.31, 3.18)	2.77 (2.44, 3.13)	2.70 (2.38, 3.06)	2.76 (2.44, 3.18)	2.82 (2.46, 3.30)	2.75 (2.42, 3.25)
G_4	-	-	2.69 (2.27, 2.98)	2.55 (2.27, 2.88)	2.65 (2.37, 3.02)	2.64 (2.34, 3.06)	2.59 (2.32, 2.92)
G_5	-	-	-	-	2.61 (2.28, 2.92)	2.70 (2.36, 3.09)	2.50 (2.27, 2.87)
G_6	-	-	-	-	-	-	2.47 (2.25, 2.83)
B_{in}	**0-3Y**	**3-6Y**	**6-9Y**	**9-12Y**	**12-15Y**	**15-18Y**	**18-24Y**
G_1	2.08 (1.87, 2.33)	2.15 (1.88, 2.39)	2.28 (1.96, 2.74)	2.65 (2.23, 3.09)	2.79 (2.30, 3.28)	2.98 (2.42, 3.54)	3.28 (2.81, 3.78)
G_2	1.95 (1.77, 2.20)	1.89 (1.66, 2.13)	2.08 (1.81, 1.40)	2.21 (1.92, 2.54)	2.29 (1.96,2.68)	2.43 (2.05, 2.90)	2.60 (2.22, 3.08)
G_3	1.62 (1.45, 2.00)	1.80 (1.57, 2.05)	2.04 (1.77, 2.40)	2.04 (1.79, 2.30)	2.11 (1.86, 2.42)	2.24 (1.92, 2.58)	2.21 (1.95, 2.60)
G_4	-	-	2.11 (1.71, 2.41)	1.98 (1.73, 2.24)	2.08 (1.83, 2.38)	2.15 (1.84, 2.49)	2.14 (1.90, 2.39)
G_5	-	-	-	-	2.02 (1.77, 2.34)	2.20 (1.83, 2.53)	2.08 (1.85, 2.35)
G_6	-	-	-	-	-	-	2.08 (1.80, 2.34)
B_{wt}	**0-3Y**	**3-6Y**	**6-9Y**	**9-12Y**	**12-15Y**	**15-18Y**	**18-24Y**
G_1	0.66 (0.51, 0.83)	0.67 (0.56, 0.82)	0.61 (0.48, 0.75)	0.55 (0.42, 0.69)	0.57 (0.43, 0.73)	0.55 (0.43, 0.69)	0.56 (0.43, 0.67)
G_2	0.57 (0.47, 0.70)	0.54 (0.42, 0.69)	0.43 (0.31, 0.54)	0.40 (0.29, 0.51)	0.40 (0.30, 0.53)	0.38 (0.28, 0.50)	0.37 (0.28, 0.48)
G_3	0.49 (0.43, 0.57)	0.47 (0.34, 0.57)	0.33 (0.25, 0.45)	0.31 (0.24, 0.42)	0.31 (0.25, 0.41)	0.29 (0.22, 0.40)	0.26 (0.21, 0.33)

G_4	-	-	0.27 (0.22, 0.34)	0.28 (0.22, 0.35)	0.29 (0.23, 0.37)	0.25 (0.20, 0.34)	0.22 (0.18, 0.29)
G_5	-	-	-	-	0.29 (0.23, 0.36)	0.26 (0.19, 0.34)	0.22 (0.18, 0.28)
G_6	-	-	-	-	-	-	0.19 (0.17, 0.25)
Artery	**0-3Y**	**3-6Y**	**6-9Y**	**9-12Y**	**12-15Y**	**15-18Y**	**18-24Y**
G_1	3.36 (2.88, 3.88)	3.43 (3.04, 3.95)	3.57 (2.94, 4.24)	3.48 (2.92, 4.14)	3.66 (3.00, 4.35)	3.68 (3.04, 4.45)	3.59 (3.09, 4.23)
G_2	3.29 (2.86, 3.91)	3.20 (2.68, 3.73)	3.06 (2.58, 3.73)	2.99 (2.52, 3.60)	3.20 (2.65, 3.83)	3.14 (2.61, 3.90)	3.00 (2.51, 3.59)
G_3	2.83 (2.44, 3.57)	3.53 (2.81, 4.25)	2.98 (2.48, 3.49)	2.75 (2.31, 3.31)	2.92 (2.43, 3.52)	2.86 (2.36, 3.49)	2.57 (2.08, 3.14)
G_4	-	-	2.85 (2.32, 3.34)	2.77 (2.34, 3.21)	2.78 (2.33, 3.36)	2.80 (2.23, 3.36)	2.32 (1.87, 2.92)
G_5	-	-	-	-	2.74 (2.34, 3.34)	2.87 (2.33, 3.46)	2.26 (1.80, 2.86)
G_6	-	-	-	-	-	-	2.59 (2.12, 3.15)

The results in each segmental generation are shown in median (IQR) by age group. IQR: interquartile range, shown in (25th percentile, 75th percentile); yrs: years; G: segmental generations, and G_{n+1} the consecutive branching bronchi; SD: standard deviation; B_{out}: bronchial outer wall diameter; B_{in}: bronchial lumen wall diameter; B_{wt}: bronchial wall thickness.

Table S3. Influence of age, sex, total lung volume, and iodine contrast on bronchial dimensions (mm)

Mixed effected models								
**B_{out}**	**Estimate**	**Std. Error**	**P-value**		**Log(B_{wt})**	**Estimate**	**Std. Error**	**P-value**
(intercept)	3.269	0.303	<0.001		(intercept)	-0.603	0.232	0.009
Age	0.020	0.007	0.005*		Age	0.009	0.005	0.087
Sex (male)	-0.095	0.042	0.026*		Sex (male)	-0.064	0.032	0.045
Total lung volume	-5.576e-05	2.053e-05	0.007*		Total lung volume	-0.0002	1.557e-05	<0.001*
Contrast (no)	-0.053	0.040	0.191		Contrast (no)	0.083	0.030	0.007*
Manufacturer(Philips)	-0.104	0.297	0.726		Manufacturer(Philips)	0.065	0.228	0.775
Manufacturer(SIEMENS)	-0.055	0.298	0.853		Manufacturer(SIEMENS)	0.118	0.228	0.605
Manufacturer(TOSHIBA)	-0.389	0.303	0.200		Manufacturer(TOSHIBA)	0.075	0.232	0.748
**B_{in}**	**Estimate**	**Std. Error**	**P-value**					
(intercept)	2.084	0.249	<0.001					
Age	0.016	0.006	0.007*					
Sex (male)	-0.026	0.034	0.455					
Total lung volume	6.348e-05	1.679e-	<0.001*					

		05						
Contrast (no)	-0.133	0.033	<0.001*					
Manufacturer(Philips)	-0.150	0.244	0.541					
Manufacturer(SIEMENS)	-0.149	0.245	0.544					
Manufacturer(TOSHIBA)	-0.466	0.249	0.062					

*B_{out}: the bronchial outer diameter; B_{in}: the bronchial lumen diameter; B_{wt}: the bronchial wall thickness; Std.Error: standard Error. *: P value less than 0.05 indicates significant difference. The reference level shows in () after the categorical variables. Note that the model of B_{wt} has log-transformed response and the coefficient (Estimate) and standard error are presented on log-scale.*

Table S4. Influence of age, sex, total lung volume, and iodine contrast on BA-ratios

Mixed effected models								
**Log(B_{out}/A)**	**Estimate**	**Std. Error**	**P-value**		**Log(B_{wt}/B_{out})**	**Estimate**	**Std. Error**	**P-value**
(intercept)	-0.018	0.130	0.889		(intercept)	-1.750	0.192	<0.001
Age	0.001	0.003	0.675		Age	0.003	0.004	0.463
Sex (male)	0.005	0.018	0.798		Sex (male)	-0.036	0.026	0.171
Total lung volume	$9.062*e^{-06}$	$8.738*e^{-06}$	0.301		Total lung volume	-0.0001	$1.286*e^{-05}$	<0.001*
Contrast (no)	-0.220	0.017	<0.001*		Contrast (no)	0.098	0.025	<0.001*
Manufacturer(Philips)	-0.007	0.127	0.957		Manufacturer(Philips)	0.084	0.189	0.656
Manufacturer(SIEMENS)	-0.084	0.128	0.509		Manufacturer(SIEMENS)	0.123	0.190	0.519
Manufacturer(TOSHIBA)	-0.118	0.130	0.364		Manufacturer(TOSHIBA)	0.198	0.193	0.305
**Log(B_{in}/A)**	**Estimate**	**Std. Error**	**P-value**		**B_{WA}/B_{OA}**	**Estimate**	**Std. Error**	**P-value**
(intercept)	-0.489	0.172	0.004		(intercept)	0.587	0.076	<0.001
Age	0.003	0.004	0.496		Age	0.0003	0.002	0.864
Sex (male)	0.025	0.024	0.294		Sex (male)	-0.017	0.010	0.109

Total lung volume	5.623*e-05	1.151*e-05	<0.001*		Total lung volume	-4.856*e-05	5.076*e-06	<0.001*
Contrast (no)	-0.265	0.022	<0.001*		Contrast (no)	0.039	0.010	<0.001*
Manufacturer(Philips)	-0.033	0.169	0.846		Manufacturer(Philips)	0.031	0.075	0.675
Manufacturer(SIEMENS)	-0.128	0.169	0.451		Manufacturer(SIEMENS)	0.047	0.075	0.532
Manufacturer(TOSHIBA)	-0.206	0.172	0.233		Manufacturer(TOSHIBA)	0.081	0.076	0.289
**Log(B_{wt}/A)**	**Estimate**	**Std. Error**	**P-value**					
(intercept)	-1.780	0.195	<0.001					
Age	0.005	0.004	0.305					
Sex (male)	-0.031	0.027	0.246					
Total lung volume	-0.0001	1.309*e-05	<0.001*					
Contrast (no)	-0.121	0.026	<0.001*					
Manufacturer(Philips)	0.078	0.191	0.682					
Manufacturer(SIEMENS)	0.039	0.192	0.840					
Manufacturer(TOSHIBA)	0.083	0.195	0.670					

*B_{out}/A: the ratio of bronchial outer diameter and adjacent pulmonary artery diameter; B_{in}/A: the ratio of bronchial lumen diameter and adjacent pulmonary artery diameter; B_{wt}/A: the ratio of bronchial wall thickness and adjacent pulmonary artery diameter; B_{wt}/B_{out}: the ratio of bronchial wall thickness and bronchial outer diameter; B_{WA}/B_{OA}: ratio between bronchial wall area and bronchial outer wall area. *P value less than 0.05 indicates significant difference. The reference level shows in () after the categorical variables. Note that the models of B_{out}/A, B_{in}/A, B_{wt}/A, and B_{wt}/B_{out} have log-transformed response and the coefficient (Estimate) and standard error are presented on log-scale.*

Table S5. Median and IQR of total lung volume, mean lung density, and Pi10 according to age group in non-iodine contrast scans

**Age range (year)**	**total lung volume (mL)**	**mean lung density (HU)**	**Pi10**
**>0 to ≤3**	441 (395, 571)	-639.45 (-694.80, -582.82)	3.46 (3.29, 3.62)
**>3 to ≤6**	1016 (931, 1182)	-715.70 (-768.60, -648.20)	3.52 (3.52, 3.54)
**>6 to ≤9**	1604 (1071, 2006)	-786.90 (-812.08, -720.35)	3.12 (2.64, 3.39)
**>9 to ≤12**	2384 (1900, 2685)	-813.85 (-833.20, -776.68)	2.73 (2.49, 2.98)
**>12 to ≤15**	3369 (2722, 4463)	-808.70 (-832.90, -775.60)	2.68 (2.45, 2.97)
**>15 to ≤18**	3778 (2978, 4418)	-804.10 (-834.00, -775.60)	2.48 (2.16, 2.79)
**>18 to ≤24**	4334 (3298, 5421)	-815.50 (-840.50, -805.40)	2.38 (1.98, 2.43)
**All**	2915 (1986, 4098)	-771.6 (-809.5, -703.0)	2.72 (2.38, 3.14)

mL: milliliter; HU: Hounsfield Unit; Pi10: the square root of wall area of a 10-mm lumen perimeter.

Table S6. Effect of age, sex, mean lung density(HU), iodine contrast, and CT scanners on total lung volume(mL)

**Regression models**								
**Mean Lung Density**	**Estimate**	**Std. Error**	**P-value**		**Pi10**	**Estimate**	**Std. Error**	**P-value**
(intercept)	-756.87	50.63	<0.001		(intercept)	5.926	0.468	<0.001
Age	7.72	1.26	<0.001*		Age	-0.025	0.009	0.006*
Sex (male)	-20.89	7.18	0.004*		Sex (male)	0.012	0.049	0.812
Total lung volume	-0.04	0.004	<0.001*		Mean lung density	0.004	0.0004	<0.001*
Contrast (no)	47.18	6.92	<0.001*		Total lung volume	$-5.869e^{-05}$	$2.880e^{-05}$	0.043*
Manufacturer(Philips)	14.01	49.06	0.775		Contrast (no)	0.007	0.050	0.896
Manufacturer(SIEMENS)	16.52	49.22	0.737		Manufacturer(Philips)	0.171	0.327	0.601
Manufacturer(TOSHIBA)	6.18	50.02	0.902		Manufacturer(SIEMENS)	0.228	0.328	0.488
**Total Lung Volume**	**Estimate**	**Std. Error**	**P-value**					
(intercept)	-6884.77	948.63	<0.001					
Age	235.39	13.34	<0.001*					
Sex (male)	-786.36	96.38	<0.001*					
Mean lung density	-9.43	0.74	<0.001*					
Contrast (no)	30.33	112.54	0.788					
Manufacturer(Philips)	15.53	731.00	0.983					

Manufacturer(SIEMENS)	193.29	733.33	0.792					
Manufacturer(TOSHIBA)	-115.22	745.22	0.877					

*Std. Error: standard error; *: P value less than 0.05 indicates significant difference. The reference level shows in () after the categorical variables.*
